# Climate change affects the suitability of Chinese cherry (*Prunus pseudocerasus* Lindl.) in China

**DOI:** 10.1186/s43897-024-00136-w

**Published:** 2025-03-06

**Authors:** Zhengxin Lv, Songtao Jiu, Li Wang, Yan Xu, Jiyuan Wang, Xunju Liu, Jieming Xu, Yuxuan Wang, Muhammad Salman Haider, Ruie Liu, Caixi Zhang

**Affiliations:** 1 School of Agriculture and Biology, Shanghai Jiao Tong University, Shanghai, 200240 China; 2 School of Life Sciences, Huaibei Normal University, Huaibei, Anhui Province 235000 China; 3 Department of Horticulture, Ghazi University, Dera Ghazi Khan, Punjab 32200 Pakistan

**Keywords:** Climate change, *P. pseudocerasus*, Biomod2, Suitability, Climate dependence, Ecological niche

## Abstract

The rapid development of *Prunus pseudocerasus* related industry has increasingly contributed to rural vitalization in China. This study employed a biomod2 ensemble model, utilizing environmental and species occurrence data from 151 *P. pseudocerasus* germplasm wild/local samples, to predict potential geographical distribution, suitability changes, climate dependence, and ecological niche dynamics. The optimized maximum entropy (MaxEnt) model yielded the most accurate predictions. The climate variables with the greatest impact on suitability were precipitation of warmest quarter and mean diurnal temperature range. The total potential suitable area for *P. pseudocerasus* was approximately 2.78 × 10^6^ km^2^, increasing with CO_2_ concentration. The highly suitable area was primarily concentrated in basin terrains, plateaus, and plains of Sichuan Province. The current centroid in Lichuan exhibited gradual latitudinal and longitudinal movement. The predicted (2090s) ecological niche trends of *P. pseudocerasus* varied under different pathways and periods, with higher CO_2_ concentration associated with lower niche overlap. The CO_2_ emission concentration in the SSP246 scenario emerged as the most suitable climate model. Climate change is driving both the expansion of geographical distribution and the contraction of overlapping geographical distribution areas of *P. pseudocerasus.* These findings provide a theoretical basis for wild resource conservation, site selection for production, and introduction of allopatry for *P. pseudocerasus*.

**Supplementary Information:**

The online version contains supplementary material available at 10.1186/s43897-024-00136-w.

## Core

The climate variables with the most significant impact on suitability were precipitation of warmest quarter and mean diurnal temperature range. The total potential suitable area for *P. pseudocerasus* was approximately 2.78 × 10^6^ km^2^, exhibiting an increase in correlation with CO_2_ concentration. The highly suitable areas were predominantly concentrated in the basin terrains, plateaus, and plains of Sichuan Province. Climate change is driving both the expansion of geographical distribution and the contraction of overlapping geographical distribution areas of *P. pseudocerasus.*

## Introduction

China has abundant germplasm resources of wild Cerasus species worldwide (Wu et al. 2003; Jiu et al. 2023, 2024a; Wang et al. 2023). Chinese cherry (*Prunus pseudocerasus*) originated in the middle and lower reaches of the Yangtze River in China and is distributed throughout the Yangtze River Basin. It is extensively cultivated in Sichuan, Guizhou, Chongqing, Henan, Shandong, Anhui, and Gansu provinces, particularly on sun-exposed mountain slopes and ravine sides (Chen et al. 2013). According to the *Flora of China*, *P. pseudocerasus* has been cultivated in China for over two millennia, making it one of the most significant and ancient cultivated fruit trees (Wu et al. 2003). *P. pseudocerasus* is highly regarded by consumers for its aesthetically pleasing tree shape, pleasant floral fragrance, vibrant fruit color, and organic nature. Consequently, it plays a crucial role in China's rural tourism industry (Chen et al. 2015, 2016).

Limited research has been conducted on the geographical distribution and ecological characteristics of these plants to enhance their production (Zhu et al. 2017, 2019). As a deciduous fruit tree, temperature plays a critical role in determining the dormancy transition of *P. pseudocerasus*. Consequently, climate change significantly impacts its distribution, and the expansion of suitable planting areas will substantially influence producers' income and industry development (Olsen, 2010). In recent years, with the growth of agricultural tourism and agriculture, *P. pseudocerasus* has garnered increasing attention due to its high ornamental value, early phenological period (flowering from February to March, and maturation from April to May), strong adaptability, and disease resistance. Consequently, the area under cherry cultivation has progressively expanded (Zhang et al. 2018; Jiu et al. 2024b).

The relationships between plants' geographical distribution patterns, climate change, and predictions of distribution changes are crucial for understanding basic ecology and biogeography, and have become a prominent research focus (Elith et al. 2009). Globally, plants’ responses to hydrothermal conditions can effectively predict their geographical distribution limits. Historical climatic conditions have shaped the current distribution pattern of *P. pseudocerasus*, which continues to be influenced by ongoing climate change. Species distributions at a regional scale are primarily affected by climate, with a combination of hydrothermal variables reflecting fundamental climatic characteristics (Woodward et al. 1987; Thuiller et al. 2011; Nadine et al. 2012). Various climatic changes exert different promoting or inhibitory effects on plants, resulting in corresponding variations in suitable areas (Camallec et al. 2024). The global climate is undergoing significant changes, substantially impacting plant geographical distribution (Kristine et al. 2013). By the end of the twenty-first century, global average surface temperature is predicted to increase by 0.3 to 4.8 ℃, accompanied by significant changes in precipitation patterns. Such dramatic climate changes may alter future geographical distribution patterns of species, exacerbate biodiversity reduction and germplasm resource loss, and potentially accelerate species extinction rates (Céline et al. 2012). Climate change significantly affects the areas, growth, development, and yield of suitable crops, as well as the prevalence of diseases and pests, and overall plant systems (Wu et al. 2023).

Mounting evidence suggests that climate warming has significantly altered agricultural climate resources in China, leading to shifts in the geographical distribution and spatial patterns of numerous crops. In Japan, climate change has modified the phenology of flowering cherry populations. Research has explored the impact of climate change on the initial four phenological stages of sweet and sour cherry trees grafted on seedlings in a temperate-continental climate. These stages include bud swelling, budburst, beginning of flowering, and end of flowering. The study also analyzed the implications for farmers. The researchers hypothesized that the onset of these phenological stages occurs earlier due to climate change (Yang et al. 2020; Primack et al. 2009; Paltineanu et al. 2020).

Climate change has significantly impacted species distribution and abundance in recent years (Thuiller et al. 2005; Alexander et al. 2015; Rumpf et al. 2019). To address research objectives, numerous scholars have introduced species distribution modeling methods (Phillips et al. 2006; Chen et al. 2011). These digital models employ algorithms to quantitatively analyze potential distribution areas or specific requirements of target species, utilizing known species distribution information and environmental variables (Phillips et al. 2006). Species distribution models (SDMs) are constructed based on specific algorithms to predict possible species distribution areas or ecological conditions of particular requirements. These models have been widely applied in various fields, including predictions of suitable area patterns and pests, as well as biodiversity monitoring (Kumar et al. 2014; Lake et al. 2020; Fang et al. 2021). SDMs have been extensively utilized in studies of species distribution, ecology, biodiversity, climate change, conservation biology, and horticultural crops (Wan et al. 2014, 2016; Duan et al. 2016; Searcy et al. 2016). For instance, Rivera-Parra et al. (2020) simulated suitable distribution areas for high-quality tea trees in Ecuador, identifying appropriate regions for Ceylon and Nilgiris. Li et al. (2020) employed the maximum entropy (MaxEnt) model and high-performance liquid chromatography to comprehensively analyze the suitability of three *Coptis chinensis* species in China. Their research indicates that the annual precipitation range and isothermality are the most significant environmental variables affecting the suitable distribution of Chinese goldthread and tea.

The ecological niche is likely a primary determinant of changes in habitat selection and spatial distribution patterns of species. Niche width indicates the range of ecological adaptation under varying carbon dioxide (CO_2_) concentration pathways and periods for *P. pseudocerasus*. Niche overlap can elucidate the similarity in ecological requirements under different CO_2_ concentrations and periods for *P. pseudocerasus*. Consequently, ecological niche analysis can predict the impact of climate change on community succession in *P. pseudocerasus* (Lasky et al. 2015). While the low information density of finite data points impedes the acquisition of high-precision spatial distribution information on temperature and precipitation, appropriate interpolation methods can effectively evaluate discrete site data to obtain precise spatial distribution information of regional precipitation (Zhang et al. 2010).

Employing the *P. pseudocerasus* fruit tree as the subject of investigation, this study utilized the MaxEnt model and a geographic information system (GIS) to simulate the suitable distribution of the tree across China. The research forecasted changes in the suitable area, area of occupancy, ecological niche, and distribution range of *P. pseudocerasus* based on climate data from various temporal periods. These findings offer a theoretical foundation for identifying suitable cherry cultivation areas and provide data-driven support for wild resource conservation, cultivation practices, agricultural industry distribution, refined planting management, and strategies to address future climate change impacts.

## Results

### Analysis of current geographical distribution and evaluation of model accuracy

**Fig. 1:**
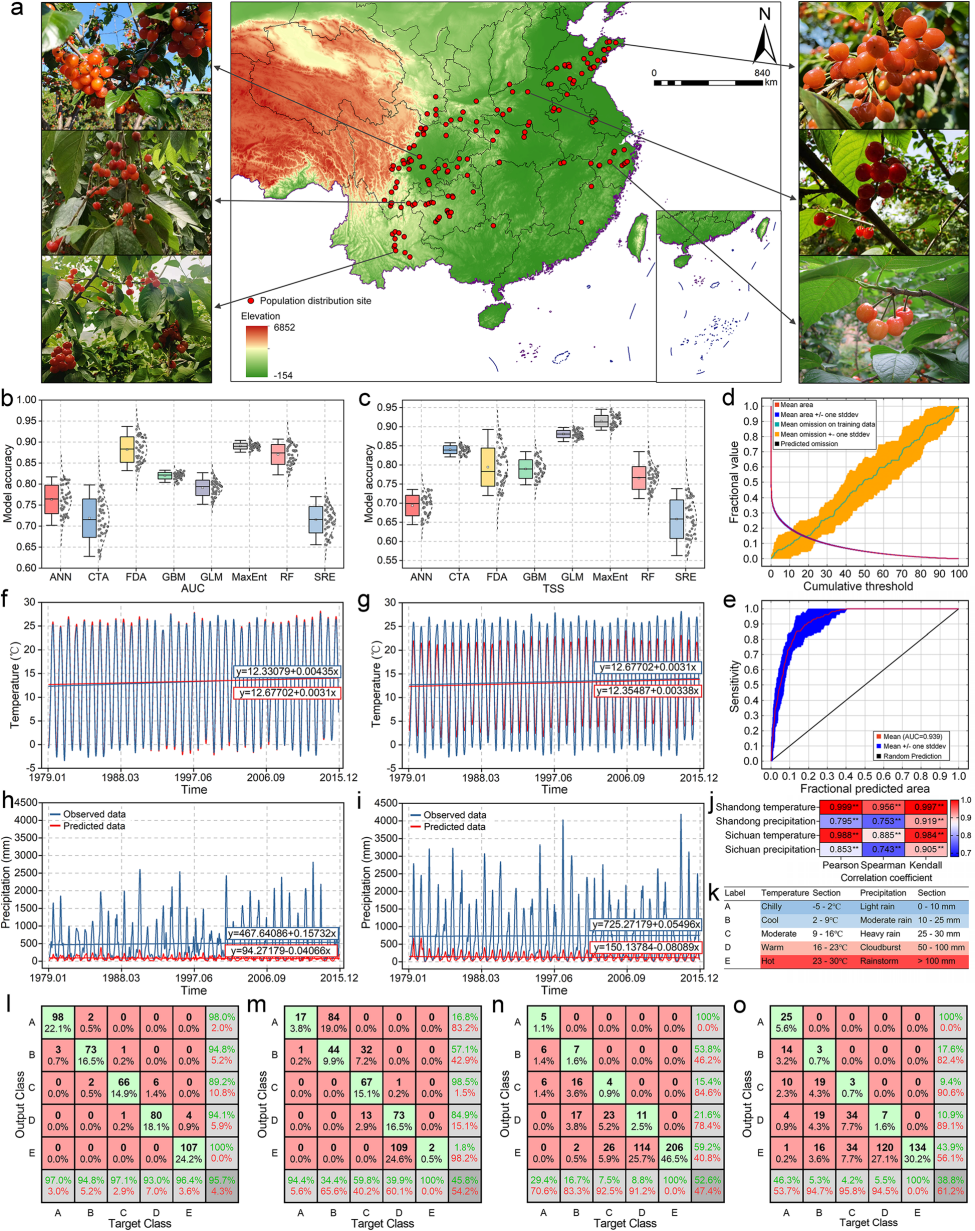
Geographical distribution evaluation indices of individual predictive models and accuracy. **a** Geographical distribution and local *P. pseudocerasus* resources. **b** AUC model accuracy test of eight climate models. **c** TSS model accuracy test of eight climate models. **d** MaxEnt model accuracy tests by fractional value. **e** MaxEnt model accuracy tests by sensitivity. **f–i** Linear regression analysis of Shandong temperature (**f**), Sichuan temperature (**g**), Shandong precipitation (**h**), and Sichuan precipitation (**i**). **j** Correlation analysis of model climate data and observed climate data. **k** Classification labels of the confusion matrix. **l–o** Confusion matrices of Shandong temperature (**l**), Shandong precipitation (**m**), Sichuan temperature (**n**), and precipitation (**o**). In **b–c**, the grey dots represent the data for each distribution. In **j**, ** indicates significance of the correlation at the 0.01 level (2-tailed). In **f–o**, temperature and precipitation data are calculated as monthly average values, with red and blue lines representing the fitted curves of observed and predicted data, respectively

The geographical distribution (151 sample points) of *P. pseudocerasus* is depicted in Fig. 1a and Table S1. Based on specimen and literature records, *P. pseudocerasus* primarily occurs in the Chinese provinces of Sichuan, Shandong, Yunnan, Zhejiang, and Guizhou. Its horizontal geographical distribution spans from 23.448°–37.356°N and 101.540°–121.828°E. The species' range extends from Yantai City, Shandong Province, in the east and north, to Liangshan Yi Autonomous Prefecture, Sichuan Province, in the west, and Mengzi City, Yunnan Province, in the south. *P. pseudocerasus* exhibits a substantial vertical distribution range (3–3488 m), with 58 collection sites recorded at altitudes exceeding 1000 m (Fig. S1a). The slope range varies from 0.007°–6.855°, and the aspect ranges from 2.251–357.022 (Fig. S1b, c).

The model accuracy test results, based on the area under the receiver operating characteristic (ROC) curve (AUC) and total sum of squares (TSS), are presented in Fig. 1b, c and Table S2. MaxEnt exhibited the highest values for both metrics (0.890 and 0.915), leading to its selection for modeling. Following the modeling process, the mean AUC value of the test data was 0.939 (Fig. 1d, e). This value exceeded the random test AUC (0.500), indicating that the ROC curve demonstrated good predictive performance for cherry distribution. To validate the accuracy of the WorldClim data and MaxEnt model, we compared real observed data with model-predicted data for Sichuan Province and Shandong Province, which had the highest number of records (35 and 30, respectively). Observed data from 1979–2015 included monthly average temperature and precipitation. We employed three analysis methods (linear fitting, correlation analysis, and confusion matrix) to verify the accuracy of these two climate indicators between observed and predicted data. Linear regression analysis revealed similar temperature data for Shandong and Sichuan provinces (Fig. 1f, g), while precipitation data showed low similarity between the two provinces (Fig. 1h, i). Correlation analysis for Shandong and Sichuan provinces indicated that Pearson's, Spearman's, and Kendall's tau-b correlations were all significant at the 0.01 level (Fig. 1j). The hierarchical labels of the confusion matrix are shown in Fig. 1k. The confusion matrix demonstrated high accuracy (95.71%) for Shandong Province's temperature data (Fig. 1l and Table S3), whereas precipitation data accuracy was only 45.84% (Fig. 1m). Both temperature and precipitation data for Sichuan Province exhibited low accuracy (Fig. 1n, o).

### Dominant environmental variables limiting *P. pseudocerasus* distribution

**Fig. 2:**
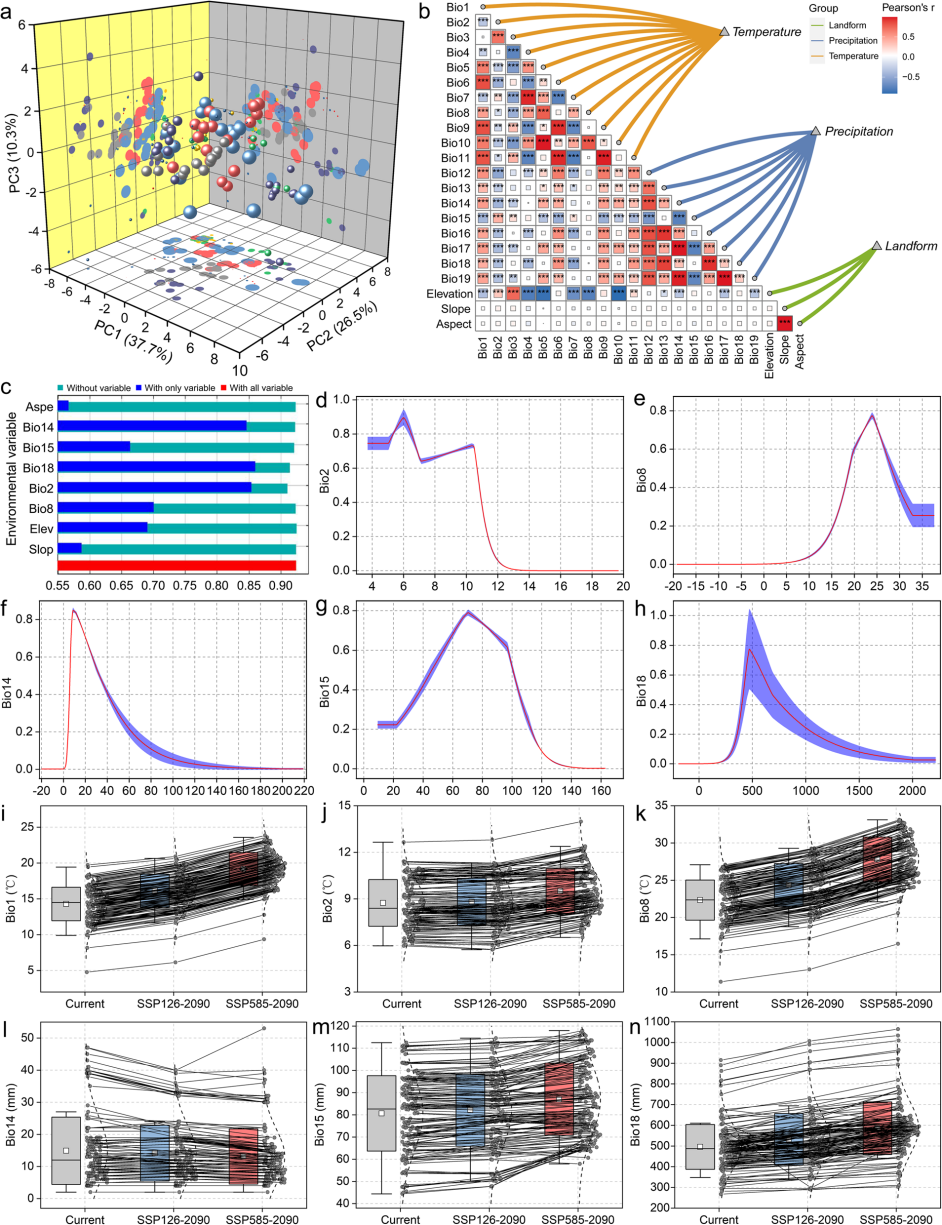
Climatic variable selection and trend analysis. **a** Principal component three-dimensional load diagram. **b** Correlation matrix heat map of 22 environment variables. **c** Jackknife test of the relative importance of environmental variables for *P. pseudocerasus* in China. **d** Response curves of Bio2, Bio8, Bio14, Bio15, and Bio18. **e** Changes in important climate variables for SSP126-2090s and SSP585-2090s. In **a**, each ball in the three-dimensional coordinate system represents an independent sample, with different colors denoting different environment variables. In **i-n**, the dashed line indicates outliers with a factor of 1, the box range represents standard deviation, the solid line in the middle of the box denotes the median, the white squares indicate averages, and the grey dots represent the data for each distribution

We screened the climate data from the distribution records and combined the principal component analysis (PCA) results to verify the data's conformity to a normal distribution. Components with eigenvalues > 1 were extracted. As shown in Table S4, the first six principal components summarized most of the original data. According to the eigenvalue and contribution rate of each eigenvector, the first principal component had the highest contribution rate, determined by Bio12, Bio1, and Bio6; the main variables were associated with hydrothermal interactions. The second principal component's contribution rate was 26.49%, determined by Bio4, Bio7, and Bio10, with the main variables associated with temperature. The third principal component's contribution rate was 10.34%, determined by Bio8, Bio1, and Bio19, also primarily associated with temperature. Three-dimensional loading plots (Fig. 2a) indicated that the environmental variables in different periods were almost on the same confidence ellipse. To avoid model overfitting, using the contribution rate of each variable obtained by PCA as one basis for screening environmental variables, we performed Spearman rank correlation testing for all data (Fig. 2b). After excluding highly correlated variables, eight variables were selected for modeling (Bio2, Bio8, Bio14, Bio15, Bio18, elevation, slope, and aspect). Furthermore, we employed Jackknife's test to assess the relative importance of the selected eight variables (Fig. 2c). The environmental variables with the highest contribution rates were Bio18, Bio2, and Bio14. These three environmental variables likely represent the dominant factors affecting the suitable habitat distribution of *P. pseudocerasus*.

The response curves depicted in Fig. 2d-h illustrate the relationship between predicted suitability and selected variables, as well as the interdependencies induced by correlations among variables. The optimal climatic conditions for *P. pseudocerasus* were identified as follows: 5–7℃ (Bio2), 20–25℃ (Bio8), 10–13 mm (Bio14), 60–80 mm (Bio15), and 480–520 mm (Bio18). Notably, all climate variables except Bio2 exhibited a similar pattern characterized by an initial increase followed by a decrease. Bio2 displayed a more complex trend. The suitability peaked when Bio2 reached 6℃, decreased between 6–8℃, slightly increased at 10.3℃, and then rapidly declined, approaching zero. Additionally, we examined the five climate variables used in the modeling under two extreme scenarios for the 2090s. Given the significant role of Bio1 in biological characteristics, we also analyzed its changes (Fig. 2i). Most variables, except Bio14, showed an upward trend, albeit with varying patterns (Fig. 2j-n). Further analysis of climate variable changes at each data point revealed some variations (Table S5). Generally, the following trends were observed: Bio8 increased with CO_2_ concentration, except in Gejiawu and Fengtong villages in Zhejiang Province. Bio14 decreased with CO_2_ concentration, but increased in Datun village (Sichuan Province), Lanzi village (Shandong Province), and Yujiahe village (Jiangxi Province). Bio18 increased with CO_2_ concentration, except in Honghuagang district and Yongyi village (Guizhou Province), and Lujiao town (Chongqing City).

### Predicted expansion and northeast shift of suitable areas of *P. pseudocerasus*

**Fig. 3:**
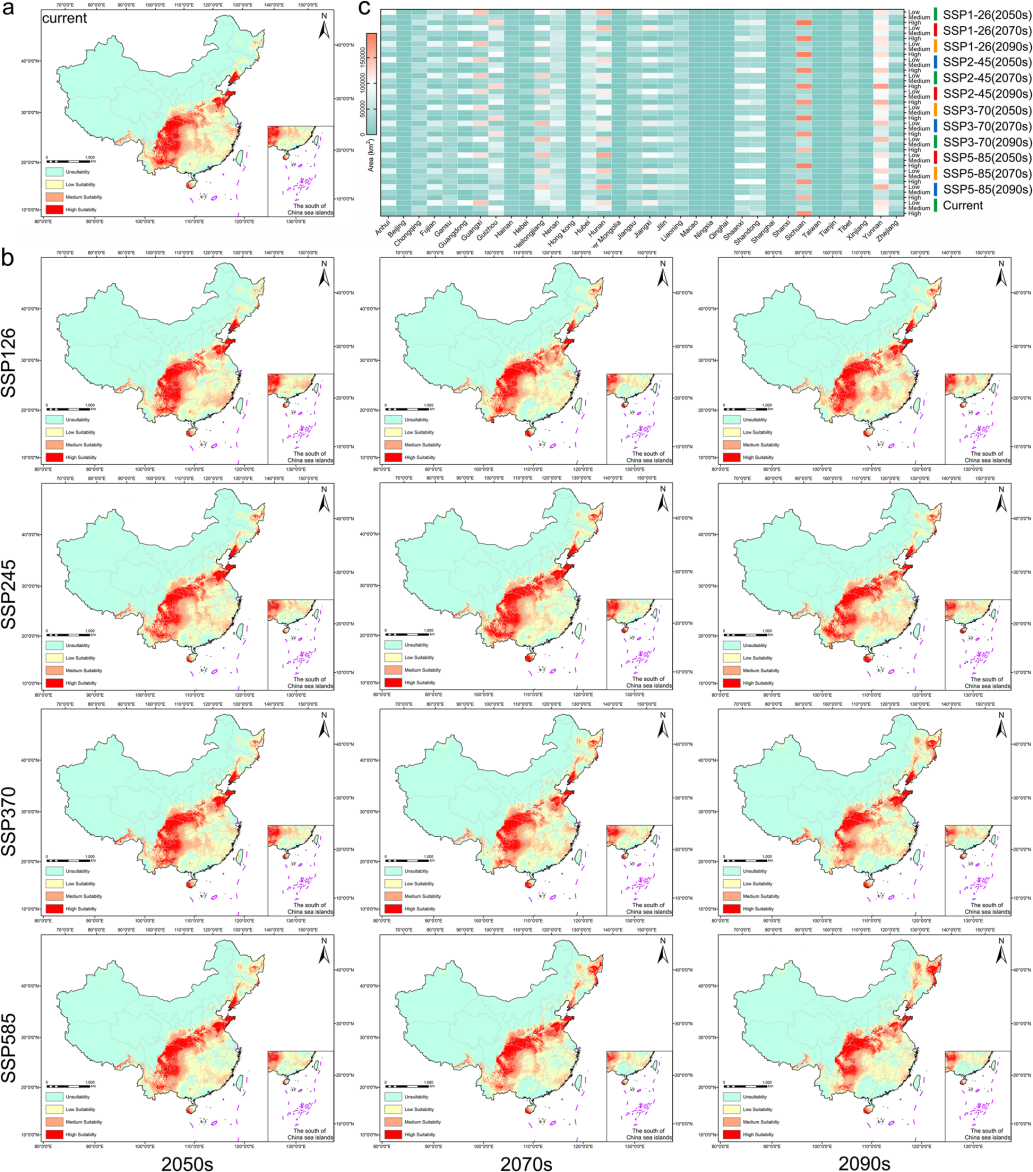
Potential distribution of the *P. pseudocerasus* cultivars. **a** Current potential distribution of *P. pseudocerasus*. **b** Potential distribution of *P. pseudocerasus* across various periods and SSPs pathways. **c** Heat map illustrating projected suitable areas for each province

The current potential distribution area of *P. pseudocerasus* encompasses Liaoning, Shandong, Tianjin, Henan, Anhui, Sichuan, Guizhou, Yunnan, Shaanxi, Hunan, Hubei, Gansu, Guangxi, and Tibet. This range extends beyond that described in the *Flora of China* (Fig. 3a). Figure 3b illustrates the changes in suitable areas across different regions during the 2050s, 2070s, and 2090s. The suitable areas consistently expand over time (Table S6). To provide a clearer representation of future changes in *P. pseudocerasus* suitability, we analyzed changes in suitable projections for provinces, cities, and autonomous regions (Fig. 3c and Table S7). Currently, the total suitable projected area is 2.78 × 10^6^ km^2^. Yunnan, Sichuan, and Hunan provinces possess the largest total suitable projected areas, while Inner Mongolia has the smallest, at zero. Across different pathways, Yunnan Province exhibited the largest suitable projected area in the 2050s, 2070s, and 2090s. However, Sichuan Province consistently demonstrated the largest high suitable projected area across all stages and pathways. Moreover, nearly all regions displayed three levels of suitability change in each period. Hunan, Qinghai, Inner Mongolia, and Hubei provinces showed insufficient areas of increased suitability, while areas of decreased and unchanged suitability were substantial. Hong Kong, Macao, and Taiwan provinces exhibited no suitability changes, although Taiwan Province experienced slight suitability decreases and remained unchanged near the north–south section of the Taiwan Mountains (Fig. 3b). Across all periods, decreased and unchanged suitabilities primarily concentrated in two regions: Shandong, northern Jiangsu, eastern Henan, and the Sichuan Basin, Yunnan Guizhou Plateau. Calculation of the sum of the three types of suitable areas (high, medium, and low) indicated that the SSP246 CO_2_ emission concentration model was the most suitable climate model for *P. pseudocerasus*.

**Fig. 4:**
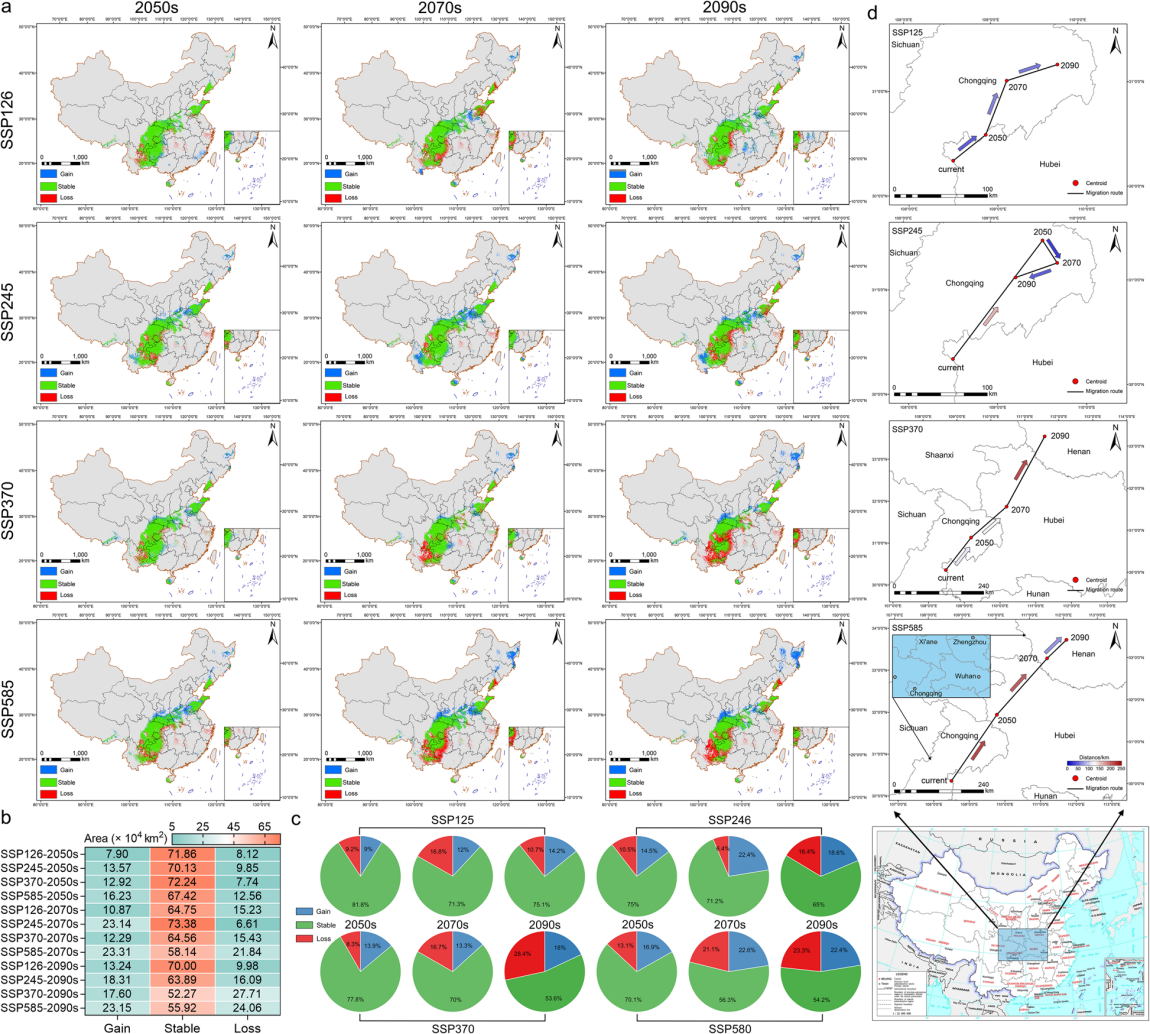
Suitable distribution and gravity center movement trajectory of the highly suitable zone under different SSPs pathways and periods. **a** Spatial distributions of suitability changes. **b** Heat map of projected area changes. **c** Proportions of gain, stability, and loss. **d** Gravity center movement trajectory of the highly suitable zone. In **d**, the arrow color indicates the magnitude of the distance (map data from http://bzdt.ch.mnr.gov.cn/)

The study compared potential distribution patterns and analyzed their spatial transformation under various future scenarios (Fig. 4a). The largest suitable gain areas were observed in the shared socioeconomic pathway (SSP585-2090s) and SSP245-2070s (Fig. 4b and Table S8). These gain rates were primarily concentrated in eastern Heilongjiang Province and southwestern Gansu Province, respectively. Conversely, SSP370-2090s and SSP585-2090s predicted the most significant loss of suitable areas, mainly in eastern Yunnan, central Guizhou, and southeastern Sichuan. The most extensive stable areas were found in SSP370-2050s and SSP126-2050s (83.49% and 82.20%, respectively), with relatively wide and fixed distributions (Fig. 4a, c). Notably, the change in suitable areas correlated with CO_2_ concentration, following a similar trend across all periods. However, the proportion of suitability change areas in each period gradually increased, suggesting that *P. pseudocerasus* may have some capacity to adapt to global warming. These suitability changes resulted in significant geographical variations. The changed areas were dispersed throughout the region, while stable areas remained relatively fixed, located primarily east of the Taihang Mountains, around the Qinling Mountains, and between the Hengduan, Daba, and Wuling Mountains (Fig. 4a).

Utilizing ArcGIS spatial analysis capabilities and MaxEnt simulation results, this study examined the migration patterns of the centroid (geometric center) of suitable cherry areas in China under climate change scenarios. The centroid of the highly suitable area was identified in Lichuan City (Enshi Tu and Miao autonomous prefecture, Hubei Province), near the Hubei-Chongqing border (Fig. 4d). The center of gravity trajectory varied across different CO_2_ emissions pathways and time periods. The most significant displacement occurred from the current position to SSP585-2050s (215.14 km), with the center of gravity shifting northeast to Zhuxi County (Shiyan City, Hubei Province). Despite variations in the moving trajectory under different pathways and periods, the predominant direction of movement was consistently northeast. Notably, the trajectories for SSP245-2050s–SSP245-2070s and SSP245-2070s–SSP245-2090s exhibited unique patterns, even moving contrary to other trajectories, possibly due to irregular climate changes during these intervals. The longitude and latitude ranges were 30.319°N–33.537°N and 108.515°E–112.046°E, respectively, with the center of gravity traversing Hubei, Chongqing, and Henan provinces. In conclusion, the analysis indicates a general trend of the center of gravity moving towards higher latitudes and longitudes (Table S9).

### Climate dependence of *P. pseudocerasus* suitability

**Fig. 5:**
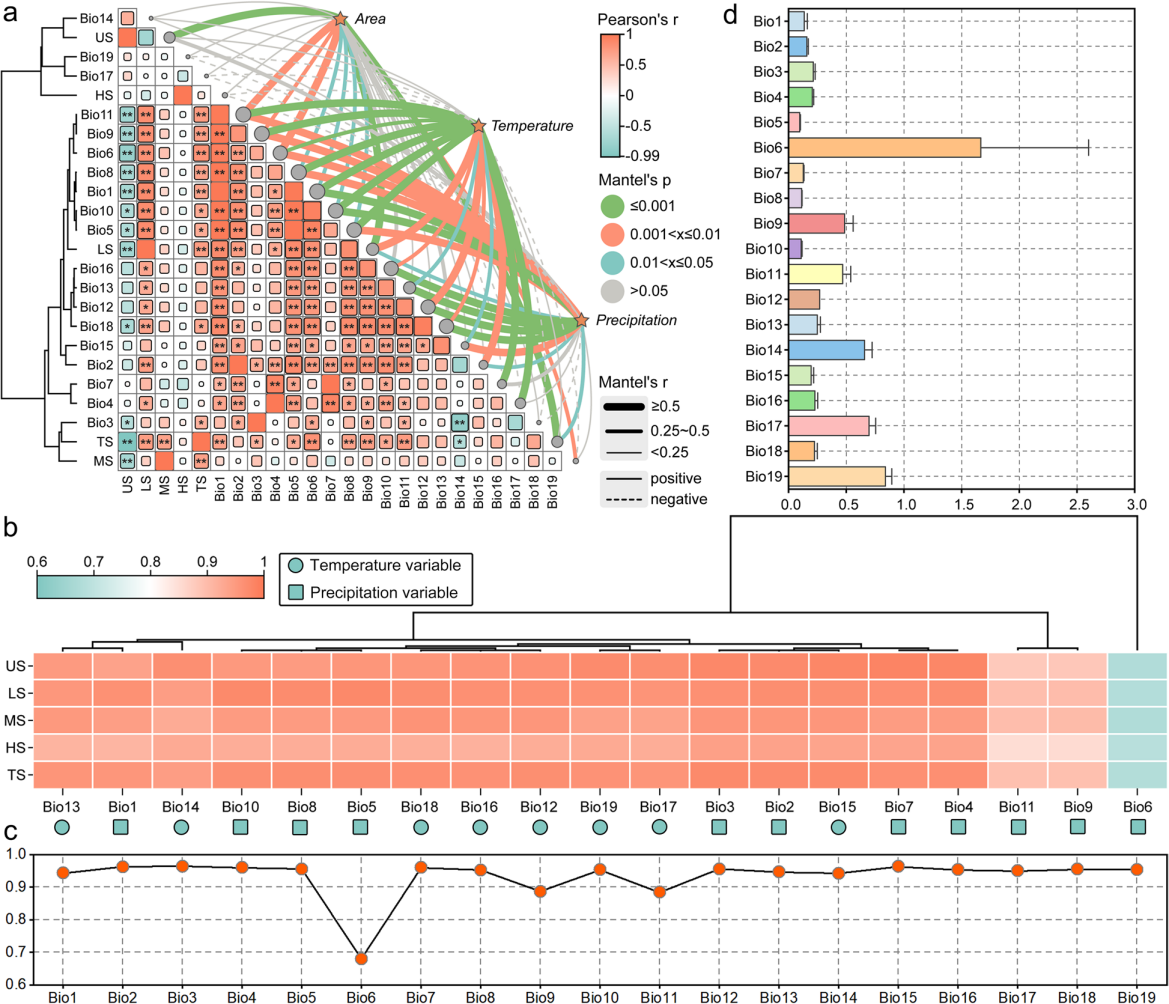
Climate dependence of *P. pseudocerasus* suitability. **a** Correlation analysis between 19 climatic variables and the five suitability area categories. **b** GRA of 19 climatic variables and the five suitability area categories. **c** Mean value of the weight for the five suitability area categories. **d** CV of all climatic variables. In **a**, ** denotes significance of the correlation at the 0.01 level (2-tailed). In **b**, US: Unsuitable area, LS: Low-suitable area, MS: Medium-suitable area, HS: High-suitable area, TS: Total suitable area. The green circles indicate precipitation variables, while green squares represent temperature variables

Climate change impacts the suitable areas for species distribution, with its influence being multifaceted, encompassing both climatic and species-specific factors. Given the comprehensive nature of climate change, to better elucidate the specific impact of one or more variables, we conducted correlation analyses between the projected areas of different suitability levels (obtained through prediction) and the climate variable data for each sample point. The results are presented in Fig. 5. The correlations varied among unsuitable, low-, medium-, high-, and total suitable areas with different climatic variables. Unsuitable areas demonstrated sensitivity to temperature variation, consistently showing negative correlations. Bio1, Bio6, Bio7, Bio8, Bio9, and Bio11 exhibited significant correlations with unsuitable areas, as did Bio3, Bio5, and Bio10. Among precipitation variables, only Bio18 showed a significant negative correlation with unsuitable areas. Low-suitable areas positively correlated with most variables. Temperature variables Bio1, Bio2, Bio5, Bio6, Bio8, Bio9, Bio10, and Bio11 showed highly significant correlations with low-suitable areas, while Bio4 demonstrated a significant correlation. Precipitation variable Bio18 was significantly correlated, and Bio12, Bio13, Bio16, and Bio18 showed significant correlations with the low-suitable area. The total suitable area also exhibited sensitivity to temperature variations, with Bio1, Bio6, Bio8, and Bio9 showing highly significant correlations, and Bio3, Bio5, and Bio10 being significantly correlated. Notably, medium- and high-suitable areas did not demonstrate significant correlations with any single climatic variable (Fig. 5a). Grey relational analysis (GRA) was employed to analyze the correlation between climatic variables and suitable areas across all periods to determine which climate variable exerts the greatest impact on the suitable area. The influences of 18 climate variables on the un-, low-, medium-, high- and total suitable areas were similar (0.851–0.984). Only the weight of Bio6 was markedly lower than those of other climate variables, with values of 0.677 for unsuitable, 0.680 for low-suitable, 0.675 for medium-suitable, 0.687 for high-suitable, and 0.679 for total suitable areas. Bio6 represents the minimum temperature of coldest month; this value suggests that the effect of low temperature on the adaptability of *P. pseudocerasus* was not as pronounced compared to that of other climate variables (Fig. 5b). The mean results of the weight for the un-, low-, medium-, high- and total suitable areas also indicated that Bio9 (0.886) and Bio11 (0.884) were slightly lower than the other variables. The remaining variables were similar (all > 0.940), except for Bio6 (0.680) (Fig. 5c). To investigate whether climate variables in different periods and sample points affect the results of correlation analysis, we analyzed the coefficient of variation (CV) of all climate variables (Fig. 5d). The CV of Bio6 was the largest among all climatic variables (1.667), it indicates a large north–south span, and also proved that low temperature was not the critical variable in adaptability of *P. pseudocerasus.*

### Ecological niche comparisons

**Fig. 6:**
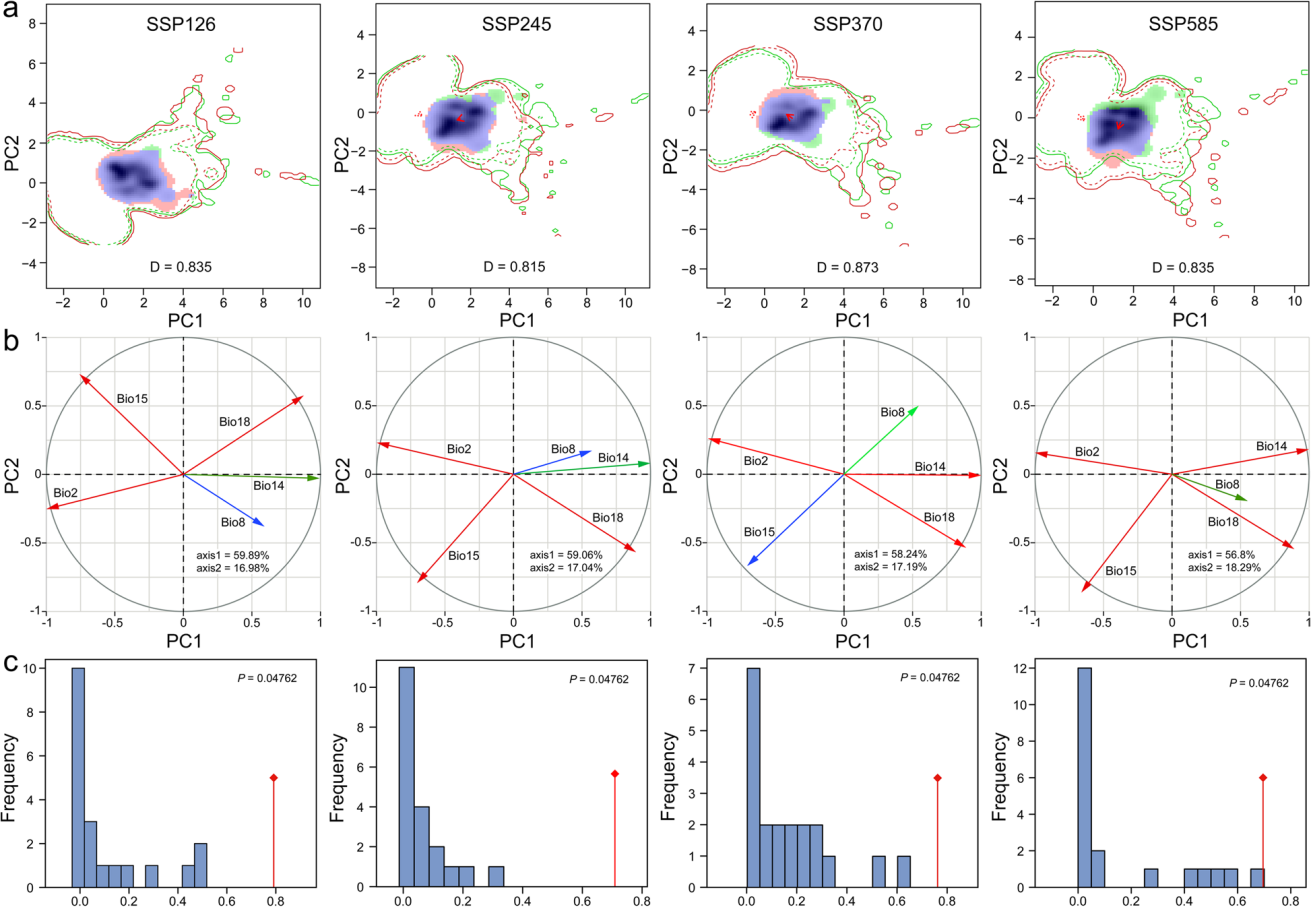
Ecological niche comparisons of *P. pseudocerasus* under different SSPs pathways in 2090s. **a** Ecological niches of *P. pseudocerasus* under SSP126–SSP585. **b** PCA load diagram for principal climate variables of ecological niches. **c** Ecological niche similarity across different pathways in 2090s. In **a**, Red arrows denote Schoener's *D*, blue indicates ecological niche overlap, green represents unfilling, and red signifies expansion. In **c**, the red arrow indicates the centroid of the realized ecological niche for each species

The ecological niche represents a species' capacity for resource utilization and adaptability, while ecological overlap indicates the state of resource competition between species. The geographical distribution of *P. pseudocerasus* is partially determined by its fundamental niche, which represents the environmental conditions necessary for its survival. Figure 6 illustrates the ecological niche dynamics of *P. pseudocerasus* in China, comparing climate niche spaces between native and invasive areas. Under each pathway, SSP585-2090s exhibited a low degree of ecological niche overlap (Fig. 6a). The ecological niches of SSP370s in invasive areas expanded compared to those in native areas. Figure 6b presents an alternative verification method for ecological niches, utilizing five climate variables for PCA to demonstrate more intuitively the ecological characteristics, revealing that different climate variables influence the niche across various pathways and periods. Table 1 presents the niche width results, indicating an increase in niche width across all pathways and periods (inverse concentration and uncertainty). These findings suggest a gradual increase in ecological adaptation. Figures S2 and S3 depict the ecological niche for the 2050s and 2070s periods. Schoener's D value greater than 0.5 indicated higher ecological niche overlap between periods under different CO_2_ emission pathways. Thus, from the perspective of ecological niche overlap, no significant changes were observed between periods. However, the degree of niche overlap decreased with increasing CO_2_ concentration. At the highest CO_2_ concentration, the niche overlap index was only 0.696 (Table S10). The null hypothesis of ecological niche similarity based on bioclimatic variables of native and invasive areas was not rejected. Figure 6c demonstrates that the null hypothesis of ecological niche similarity based on different CO_2_ emission pathways was not rejected (*P* = 0.04762, *P* < 0.05). As a species native to China, the evolution of its ecological niche aligned with expectations. Both D (D > 0.7) and I (I > 0.9) indices suggested a high degree of ecological niche overlap between different pathways and years. The ecological niches of different pathways and years may be similar but not identical.

**Table 1:** Niche width between different pathways and periods of *P. pseudocerasus*

	B1 (Inverse concentration)	B2 (Uncertainty)
Current	0.223825822	0.904068475
SSP126-2050	0.230269365	0.906669957
SSP245-2050	0.244686155	0.910713887
SSP370-2050	0.238489968	0.909349337
SSP585-2050	0.239890593	0.91059111
SSP126-2070	0.227854272	0.906509024
SSP245-2070	0.246757808	0.910857592
SSP370-2070	0.245541849	0.912495324
SSP585-2070	0.244777952	0.912301375
SSP126-2090	0.241194769	0.909243294
SSP245-2090	0.243854752	0.910729913
SSP370-2090	0.244257411	0.913615245
SSP585-2090	0.245998768	0.91335911

### Degree of climate anomaly and spatial interpolation inversion

The mean multivariate similarity across the four future climate change scenarios ranged from 6.57 to 17.24. The SSP126-2050s climate scenario exhibited the highest multiple similarity and the lowest degree of climate anomaly, while the SSP585-2090s scenario demonstrated the lowest multiple similarity value and the highest degree of climate change (Figs. S4 and S6). As CO_2_ emission concentrations increased for the same time period, the mean multivariate similarity value decreased (Figs. S4-6 and Table S11). At the locality level, the range spanned from −0.25 to 54.23. The lowest value was observed in Taihe County, Fuyang City, Anhui Province (SSP585-2090s), while the highest was recorded in Qinzhou District, Tianshui City, Gansu Province (SSP126-2090s). These differences primarily stemmed from variations in climate, altitude, geographic location, and terrain. In the modern suitable habitats for *P. pseudocerasus*, the most dissimilar variables were the mean temperature of the wettest quarter, mean diurnal range, precipitation in the warmest quarter, precipitation seasonality, and precipitation in the driest month. Precipitation variables dominated the vast majority of the adaptive area, followed by temperature variables. Terrain variables accounted for a negligible portion of the adaptation area.

The spatial interpolation results for *P. pseudocerasus* in Shandong and Guizhou are presented in Fig. S7. The timescale data were transformed into a spatial scale to illustrate the spatial distribution of various climate variables. All climate variables demonstrated similar current and future distribution patterns, with a notable spatial migration strongly correlated with climate change (Tables S3 and S5). We computed the error value and mean square error value for the current period and the 2090s (Fig. S7, Tables S12, 13). The mean square error range for most variables fell between −10 and 15 (Fig. S8). Notably, under the SSP585 pathway, Bio18 exhibited the largest error value among the five climate variables in Shandong Province for the 2090s. This discrepancy may be attributed to the uneven distribution of the distribution records used in the interpolation. In summary, Guizhou Province displayed smaller interpolation errors, while Shandong Province demonstrated greater irregularity, potentially due to its more complex geographical environment.

## Discussion

### Potential distribution area of *P. pseudocerasus* and reliability of MaxEnt prediction

This study predicted potentially suitable areas for *P. pseudocerasus* and analyzed the effects of 22 environmental variables on their growth and distribution. Yunnan, Sichuan, and Hunan provinces emerged as the most suitable distribution areas in China (Fig. 1a), aligning with records in *Flora of China* and other sources. Taiwan Province exhibited low- and medium-suitability areas among the current potentially suitable regions after simulation. Although reliable geographical information on *P. pseudocerasus* in Taiwan was not found in relevant literature, this finding corroborates the actual situation (Wu et al. 2003). The model accuracy test method employed in this study was the AUC method, with values ranging from 0.5 to 1. Values approaching 1 indicate higher accuracy. The AUC value of the test and training data was 0.927 and 0.928, respectively, suggesting high reliability of the selected geographic information (Phillips et al. 2006). As *P. pseudocerasus* is a large woody plant, it possesses a wider niche and higher degree of conservation compared to herbaceous and dwarf shrubs. Consequently, this study utilized the MaxEnt model to simulate the potential distribution of *P. pseudocerasus*. However, MaxEnt has limitations in considering biological interactions, tending towards a conservative niche. Overly complex models may present challenges in ecological interpretation. To mitigate this error, sample deviation processing and variable correlation analysis were employed to enhance the model's prediction accuracy. Nevertheless, the default parameters in MaxEnt were not adjusted according to species characteristics, potentially introducing errors and uncertainties that warrant further investigation to refine the model.

Model verification is a crucial final step in the modeling process, as accuracy directly determines data reliability (Deng et al. 2022). In this study, model accuracy was assessed using the ROC response curve. Actual climate data from the two provinces with the most extensive species distribution were utilized to compare similarity and accuracy with the model data. The precipitation data accuracy for Shandong and Sichuan provinces was low (< 50%) based on the confusion matrix (45.8% and 38.8%, respectively). These results may be attributed to the confusion matrix being a fuzzy-index evaluation method. Additionally, the classification standard of precipitation levels by the China Meteorological Administration, used as the label in this study, may contribute to this outcome (Deng et al. 2016). Precipitation is influenced by complex variables, and models capable of accurately predicting future precipitation have not yet been developed, despite precipitation being a critical environmental factor affecting species distribution and migration. Nevertheless, based on high-precision temperature data, linear regression analysis, and correlation analysis, we conclude that the model simulation results are more accurate.

### Environmental variables influence the geographical distribution of *P. pseudocerasus*

Environmental conditions significantly influence plant growth and development, serving as primary determinants of species distribution (Thuiller et al. 2007). Following PCA and correlation analysis of 22 environmental variables, eight variables were identified as having the most substantial impact on the geographical distribution of *P. pseudocerasus*. These variables encompassed temperature, precipitation, and geographical factors. Previous MaxEnt-based studies on species distribution in China have demonstrated that these eight climate variables also exerted significant influence on the geographical distribution of other plant species, including *Ulmus lamellosa*, *Scutellaria baicalensis*, and *Dalbergia cultrata* Graham ex Benth (Yan et al. 2017; Liu et al. 2019; Xu et al. 2020 ; Zhao et al. 2018). Isothermality exhibited a positive correlation with mean diurnal temperature range (Bio2) and a negative correlation with the annual temperature range (Bio7). Optimal suitability was observed when the mean temperature of the wettest quarter (Bio8) ranged from 20–25℃, indicating that extreme temperatures, whether high or low, can diminish adaptability (Zhou et al. 2023). Excessively high temperatures adversely affect pistil and stamen development in *P. pseudocerasus*, potentially leading to abortion (Li et al. 2010a, b), while excessively low temperatures reduce cell viability, delay development, and may cause frostbite (Wang et al. 2019; Guy et al. 2008). Interestingly, the CV of minimum temperature of coldest month (Bio6) was the largest among all climate variables, it indicates that *P. pseudocerasus* was less sensitive to low temperature and more cold-tolerant. The phenological period of *P. pseudocerasus* precedes that of sweet cherry, with flowering typically occurring from February to April, new shoot growth peaking from February to May, and fruit ripening from April to May. However, due to significant geographical variations, the phenological period can vary considerably (Li et al. 2010a, b; Wang et al. 2022). Most plants undergo a warm-cold-warm stage during their growth and development periods. Consequently, temperature variations (mean diurnal range, daily temperature difference, and annual temperature difference) likely represent one of the most critical variables affecting plant growth and development (Fadón et al. 2019).

The contribution rates of precipitation in the wettest month (Bio18) and driest month (Bio14) were the most significant among all climate variables. These variables influence the geographical distributions of *Alternanthera philoxeroides*, *Chromolaena odorata,* and *Lantana camara* (Yan et al. 2020; Saranya et al. 2021). Adequate water facilitates plant growth and development during peak periods. However, *P. pseudocerasus* exhibits poor waterlogging tolerance. Excessive soil water content can compromise tree vigor and potentially lead to mortality. Furthermore, excessive precipitation during maturation directly results in fruit cracking (Cui et al. 2009). Fruit cracking in *P. pseudocerasus* has not received substantial attention, although it suggests that excessive rainfall reduces environmental adaptability (Suran et al. 2019). Analysis of precipitation in the potential growth areas revealed that despite differences in topography and climate types in the Sichuan Basin, Yunnan-Guizhou Plateau, North China Plain, northern Jiangsu Plain, and Taiwan Mountains, common characteristics were observed. Notably, more than 50% of the annual rainfall is concentrated in the summer, coinciding with fruit maturity (Chen et al. 2015, 2016) and generally aligning with the biological habits of *P. pseudocerasus*. Climate variability is evident, with precipitation seasonality (Bio15) having the third-highest water variable contribution rate, correlating strongly with the rainfall characteristics of the current potential distribution areas. The variance positively correlated with seasonal variations in precipitation fluctuations. Within a certain range, greater variance indicates better adaptability.

Climate variables significantly influence geographical distribution patterns by regulating the growth and development of species. Hydrothermal variables, in particular, play a crucial role in shaping ecological adaptation. These findings align with studies conducted on other species (Lu et al. 2018; Zhao et al. 2018). Climate change primarily disrupts the fit between species' adaptive traits and their environment. However, plants have evolved mechanisms to cope with climate-related disturbances (Aitken et al. 2008). In the case of *P. pseudocerasus*, further research is necessary to investigate the application of climate scenario simulations using various climate system models and to conduct comparative analyses of habitat differences in Chinese cherry populations.

### Changes in distribution patterns and protection and management of *P. pseudocerasus*

The analysis of potential distribution areas in the current and future periods revealed that the present highly suitable areas for *P. pseudocerasus* primarily encompass the eastern Himalayas (southeast of the Tibet Autonomous Region), Hengduan Mountains (at the junction of eastern Sichuan, Yunnan, and Guizhou), Shandong, northern Jiangsu, and eastern Henan. The simulations for suitable areas in the 2050s, 2070s, and 2090s demonstrated minimal changes compared to the geographical distribution and current potential suitable areas. These areas remained predominantly concentrated around the Qinling Mountains-Huaihe River, with highly suitable regions primarily at the eastern and western extremities. The suitable regions encompass diverse terrains, including the Sichuan Basin, Yunnan-Guizhou Plateau, North China Plain, and Jianghuai Plain. These areas provide adequate atmospheric and thermal conditions, and their numerous mountains offer a relatively stable environment for plants during this period of climate fluctuation (Jordi et al. 2011).

Geographical and climatic variables, including landform, altitude, precipitation, and temperature, revealed the high niche diversity of *P. pseudocerasus* in China and its capacity to adapt to diverse environments with significant variations in climate, terrain, and altitude. The findings indicate the robust climate adaptability of *P. pseudocerasus*. Analysis of potential distribution areas across three different periods demonstrated that the total suitable area did not change substantially, possibly due to the absence of significant climate change. Most species exhibit niche conservatism and thus tend to inhabit areas with the most favorable climatic conditions. Consequently, a lesser degree of climate change would result in less pronounced alterations in species distribution (Parmesan et al. 1996). The potentially suitable habitat for this species has experienced considerable reduction in areas with minor topographic and geomorphic changes.

In recent years, global warming, driven by increased greenhouse gas emissions and a marked rise in extreme weather events, has significantly impacted the habitats of numerous organisms (Barnaud et al. 2010; Hirabayashi et al. 2022; Wu et al. 2022). Globally, species migration generally exhibits a trend towards higher altitudes and latitudes. However, the SDM results indicate that the potential future distribution area of *P. pseudocerasus* does not demonstrate a clear pattern of migration to higher altitudes. This observation may be attributed to either minimal climate change in its habitat or the species' long evolutionary history and successful adaptation to its current ecological niches (Shi et al. 2014). Consequently, future research should incorporate additional variables, such as soil characteristics, growth patterns, and anthropogenic influences, to enhance model accuracy and provide a more comprehensive projection of the future distribution of *P. pseudocerasus*.

To enhance the classification of climate variable changes across different pathways and periods, we conducted a cluster analysis of 151 distribution records based on five selected climate variables. The results are presented in Fig. S9. The Ward method was employed to categorize the distribution records into five distinct groups. The clustering outcomes for the current and 2090s periods demonstrated remarkable consistency in terms of group numbers, distances, and members. Notably, geographical proximity did not necessarily correlate with similar clustering results for all sampling points. Some geographically distant sampling points exhibited close clustering distances. While sampling points within the same province were partially clustered, others were dispersed, particularly in Sichuan and Shandong. This pattern suggests two key insights: firstly, it indicates high terrain and climate diversity in these regions; secondly, it points to significant climate change occurring in these areas.

*P. pseudocerasus* has an extensive cultivation history in China, yet only a limited number of wild resources have been commercially cultivated for an extended period. The slow promotion rate in China is attributed to the challenges associated with long-distance transportation after the color-changing stage. To develop commercial *P. pseudocerasus* cultivars, increased protection should be afforded to wild resources, and efforts should be made to artificially domesticate high-quality wild accessions. Elucidating the ecological characteristics of *P. pseudocerasus* will facilitate its introduction into suitable areas for adaptive cultivation, thereby promoting and preserving *P. pseudocerasus* resources in China.

## Conclusion

The MaxEnt model provided geographical distribution information of *P. pseudocerasus* and corresponding environmental variable data. This study predicted that the primary potential distribution areas for cherry cultivation in China are Yunnan, Sichuan, and Hunan provinces. These regions are considered suitable for commercial cultivation. Additionally, most areas of Shandong Province and Dalian City were identified as highly suitable for *P. pseudocerasus* growth. Among the climate models examined, the SSP246 CO_2_ emission concentration scenario yielded the largest total suitable area for *P. pseudocerasus*. While the 22 environmental variables selected in this study offer valuable insights, they may not comprehensively represent all climatic factors influencing the geographical distribution of *P. pseudocerasus*. Hydrothermal conditions, abiotic factors (such as light, air, and soil), species interactions, and migration ability significantly impact species distribution. Future research should incorporate a broader range of biological and abiotic factors during model construction to enhance prediction reliability. This study elucidates the response of *P. pseudocerasus* geographical distribution patterns to climatic variables, providing a scientific foundation for the conservation, development, and utilization of wild germplasm resources.

## Materials and methods

### Species geographical distribution data

The geographical distribution records of *P. pseudocerasus* were compiled and analyzed based on the checklist of SDMs. Initially, the geographical distribution data of *P. pseudocerasus* were sourced from multiple databases: the Chinese Virtual Herbarium (http://www.cvh.org.cn), National Specimen Information Infrastructure (NSCII, http://www.nsii.org.cn), Teaching Specimen Resource Sharing Platform (http://mnh.scu.edu.cn), Global Biodiversity Information Facility (https://www.gbif.org/), and *Flora of China* (http://www.iplant.cn/foc). Subsequently, duplicate distribution records and those lacking detailed geographical information were eliminated. The longitude and latitude of distribution records at different locations were then converted into actual projected linear distances. Lastly, distribution records with intervals < 5 km were removed. The resulting 151 distribution records of *P. pseudocerasus* are presented in Table S1.

### Environmental variables

Raster files for 22 bioclimatic variables (19 climate and three digital elevations) with a resolution of 5 arc minutes were obtained from WorldClim (v2.1, https://worldclim.org/). Future climate and digital elevation data were acquired from the WorldClim database (http://www.worldclim.org//; accessed 2023). The Climate Model Intercomparison Project 6 (CMIP6), which offers significant advantages in climate simulations, was selected. Shared Socioeconomic Pathways (SSPs) were chosen for climate data during the 2050s, 2070s, and 2090s. Atmospheric CO_2_ content is the primary factor influencing global temperature. CMIP6 was selected due to its high accuracy in predicting changes in CO_2_ concentration. It is a universal climate system model applicable to most terrestrial plants. Human emissions of CO_2_ and other greenhouse gases are the main drivers of climate change (Mitchell et al. 2000). Using the provincial administrative division vector map as the base map, data for the 22 variables for the three periods were cropped in ArcGIS to obtain the climate layer for the corresponding period. To determine the geographical distribution of suitable habitats for a species, it is crucial to identify the environmental variables affecting species growth. The environmental variables and their definitions are listed in Table 2.

**Table 2:** The 22 environmental variables of this study

enviromental variables	enviromental variables
Bio1 Annual Mean Temperature	Bio12 Annual Precipitation
Bio2 Mean Diurnal Range	Bio13 Precipitation of Wettest Month
Bio3 Isothermality	Bio14 Precipitation of Driest Month
Bio4 Temperature Seasonality	Bio15 Precipitation Seasonality
Bio5 Max Temperature of Warmest Month	Bio16 Precipitation of Wettest Quarter
Bio6 Min Temperature of Coldest Month	Bio17 Precipitation of Driest Quarter
Bio7 Temperature Annual Range	Bio18 Precipitation of Warmest Quarter
Bio8 Mean Temperature of Wettest Quarter	Bio19 Precipitation of Coldest Quarter
Bio9 Mean Temperature of Driest Quarter	Elevation
Bio10 Mean Temperature of Warmest Quarter	Slope
Bio11 Mean Temperature of Coldest Quarter	Aspect

### Analysis of dominant environmental variables

The ArcGIS software's Extract Multi-values to Points tool was employed to extract environmental variable information from the 151 distribution records. Due to the correlation among environmental variables, it was necessary to select and remove certain variables to prevent overfitting of the species distribution model. PCA and Spearman rank correlation tests were conducted using SPSS v.24.0 software on the environmental variables. Variables with high variance contribution rates were identified, and correlation coefficients were compared to eliminate variables with high correlations (|R|> 0.7). In instances where two variables exhibited a high correlation, the variable with the higher contribution rate was retained.

### Suitable habitat distribution model

Biomod2 (BIOdiversity MODelling2), utilizing distribution point data and environmental variables, predicted suitable habitat distribution. After evaluating all models' accuracy through AUC and TSS values, the optimal model was selected for further analyses. The contribution of each environmental variable was determined using PCA (Akomolafe et al. 2020). Response curves were established to obtain logistic relationships between distribution probability and environmental variables. The ROC curve was employed to assess the model's prediction accuracy, with the random test percentage set at 25, model replicates at 10, and remaining parameters at default values (Segurado et al. 2004). ArcGIS software was utilized for post-processing simulation results, including hierarchical displays, area statistics, and centroid calculations (Yan et al. 2021). Following predictions and analysis using the MaxEnt model, the potential distribution areas of *P. pseudocerasus* were categorized into four grades using the natural segmentation method. The suitable habitat class of *P. pseudocerasus* was determined based on the *P* value, where *P* < 0.2 indicated unsuitable areas, 0.2 ≤ *P* < 0.5 denoted low-suitable areas, 0.5 ≤ *P* < 0.7 represented medium-suitable areas, and *P* ≥ 0.7 signified high-suitable areas (Xu et al. 2019; Sharifian et al. 2021).

### Model accuracy test

The model evaluation was conducted by inputting the acquisition point distribution data and environmental variable layers into MaxEnt. The accuracy of the suitable area distribution predicted by MaxEnt was assessed using the ROC curve, from which the AUC was derived. The AUC values range from 0.5 to 1, with values closer to 1 indicating higher accuracy (Swets et al. 1988). The interpretative standards for AUC values were as follows: 0.50–0.60 (fail), 0.61–0.70 (poor), 0.71–0.80 (fair), 0.81–0.90 (good), and 0.91–1.00 (excellent). The confusion matrix analysis was performed using MATLAB R2022b, employing the following calculation formulae:

$$\left\{\begin{array}{l}\mathrm{YValidatio}\;\mathrm n\;=\;\mathrm{data}.\;\mathrm{VarName}2;\\\mathrm{YValPred}=\mathrm{data}.\;\mathrm{VarName}2;\\\mathrm{Plabels}=\mathrm{categorical}(\mathrm{YValPred});\\\mathrm{Alabels}=\mathrm{categorical}(\mathrm{YValidat}\;\mathrm{ion});\\\mathrm{plotconfus}\;\mathrm{ion}(\mathrm{alabel}\;\mathrm s,\;\mathrm{plabels});\end{array}\right.$$

The extreme values derived from temperature and precipitation data were categorized into five classes for subsequent analysis. Historical climate data were obtained from various weather stations, while model climate data were acquired from the National Tibetan Plateau / Third Pole Environment Data Center (https://data.tpdc.ac.cn/home).

### Measurement of ecological niche and spatial interpolation

To enhance understanding of the influence of CO_2_ concentration and temporal factors on environmental variables, we integrated the MaxEnt modeling results to obtain spatial interpolation data. Thin plate smoothing spline was employed to invert spatiotemporal changes in environmental variables. The ecospat package in R Studio 4.1.3, utilizing PCA-env and centroid shift, overlap, unfilling, and expansion schemes, was implemented to analyze ecological niches (Broennimann et al. 2012; Guisan et al. 2014; Di et al. 2017). Relevant bioclimatic variables were used to perform PCA-env. Subsequently, the ecospat package was utilized to conduct climate niche similarity tests, performed bidirectionally with 1000 repetitions. Enmtools1.3 was employed to determine niche differences between various CO_2_ concentration pathways and periods, including niche overlap and width. In the ecological niche overlap test, Schoener's *D* and Hellinger's distance (*I*) were utilized to quantify similarities between the ecological niches of the three periods and to compare the degree of ecological niche overlap in their native areas (Xian et al. 2023). The values of *D* and *I* ranged from 0 (no overlap) to 1 (> 0.6 indicating significant overlap).

### Analyses of multivariate environmental similarity surface and most dissimilar variable

Utilizing the environmental variables in the current potential distribution area (101.5395°–121.8275°E, 23.4476°–37.3556°N) of *P. pseudocerasus* as the reference layer, a multivariate environmental similarity surface was employed to assess the degree of climatic anomaly in the current suitable habitats under past and future climate change scenarios. The most dissimilar variable was analyzed to identify the key factors driving changes in the potential geographical distribution. The multivariate environmental similarity surface calculated the similarity (S) between environmental variables under future climate conditions and contemporary environmental variable point sets to determine the extent of environmental change in the distribution area. The environmental variable with the lowest S-value (highest degree of abnormality) at a point was considered the least similar variable at that location. Environmental variables exhibiting the highest levels of anomalies were likely to be the primary factors causing shifts in the distribution of suitable habitats. This analysis was conducted using the ‘density.tools.Novel' command in the ‘maxent.jar' file.

### Migration of the center point of suitable area

The SDMTool package in R software was employed to analyze the trends in suitable habitat regions and the geometric center positions of these areas for the current period, as well as the 2050s, 2070s, and 2090s. The suitable habitat was considered as a single entity and reduced to a vector particle, utilizing the centroid position changes to reflect the size and direction of the species' suitable habitat. Subsequently, the centroid was tracked using various SDMs to examine its position across different time periods and under varying climatic conditions. This approach facilitated the evaluation of the suitable zone's migration distance in terms of latitude and longitude coordinates.

### Statistical analyses

Statistical analysis was conducted using Microsoft Excel 2020. SPSS v.24.0 was employed for the PCA and best-fit analysis, while Pearson's method was utilized for linear and partial correlation analyses. PCA was performed to explore correlations among variables and 151 distribution records, determine maximum or minimum correlation directions, and achieve data compression to enhance result accuracy (Abdi et al. 2010). Data fitting was conducted using a linear regression equation based on the discrete form of the data (Zhao et al. 2022). Additionally, GRA was employed to analyze 19 climate variables to determine which had a greater impact on suitable areas. To more intuitively understand the relationship between correlated climate variables and suitability, we analyzed the variability of climate variables across different periods to demonstrate the impact of fluctuations on suitability. Microsoft Excel 2010 was used for CV analysis. Ward's method, Euclidean distance, and sum of distances cluster analyses were performed using Origin Pro 2022 software (Xie et al. 2020). Analysis of variance and Duncan's multiple range test were conducted to determine significant (*P* = 0.05) differences between groups. The ggcor package in R software was employed to analyze the correlations of variables and projected areas. Trends and figures within the data were graphically depicted using OriginPro 2022 and RStudio. All data used for correlation analysis and PCA underwent data standardization.

## Supplementary Information

Additional File 1: Supplement Table S1. Locality data for this study (151 distribution records). Additional File 1: Supplement Table S2. Eight models of biomod2 test results. Additional File 1: Supplement Table S3. Raw data of confusion matrices. Additional File 1: Supplement Table S4. PCA data of the 22 variables. Additional File 1: Supplement Table S5. Extracted data from 6 climatic factors at 151 sample points. Detailed data of centroid displacement. Additional File 1: Supplement Table S6. Area of each suitability category. Additional File 1: Supplement Table S7. Suitable area for each province under different pathways and time periods. Additional File 1: Supplement Table S8. Changes in area suitability during different pathways and periods. Additional File 1: Supplement Table S9. Detailed data of centroid transfer. Additional File 1: Supplement Table S10. Niche overlap index in different periods and SSPs pathways. Additional File 1: Supplement Table S11. The mean value of multivariate similarity. Additional File 1: Supplement Table S12. Raw data of spatial interpolation inversion for Shandong Province. Additional File 1: Supplement Table S13. Raw data of spatial interpolation inversion for Guizhou Province.

Additional File 2: Supplement Figure S1. The landform with 151 distribution records of *P. pseudocerasus*. a Elevation. b Slope. c Aspect. Additional File 2: Supplement Figure S2. Ecological niche comparisons of *P. pseudocerasus* under different pathways in 2050s. a Ecological niches of *P. pseudocerasus* with SSP126-2050s–SSP585-2050s. Red arrows indicate Schoener's D. Blue indicates ecological niche overlap, green indicates unfilling, and red indicates expansion. b Alternative verification method of ecological niches with SSP126-2050s–SSP585-2050s. c Ecological niche similarity of different pathways in 2050s. The red arrow indicates the centroids of each species' realized ecological niche. Additional File 2: Supplement Figure S3. Ecological niche comparisons of *P. pseudocerasus* under different pathways in 2070s. a Ecological niches of *P. pseudocerasus* with SSP126-2070s–SSP585-2070s. Red arrows indicate Schoener's D. Blue indicates ecological niche overlap, green indicates unfilling, and red indicates expansion. b Alternative verification method of ecological niches with SSP126-2070s–SSP585-2070s. c Ecological niche similarity of different pathways in 2070s. The red arrow indicates the centroids of each species' realized ecological niche. Additional File 2: Supplement Figure S4. Multivariate environmental similarity surface and most dissimilar variable analysis under different combinations of climate change scenarios in 2050s. a Multivariate environmental similarity surface and most dissimilar variable in 2050s. b Heat map of multivariate environmental similarity surface area in 2050s. Additional File 2: Supplement Figure S5. Multivariate environmental similarity surface and most dissimilar variable analysis under different combinations of climate change scenarios in 2070s. a Multivariate environmental similarity surface and most dissimilar variable in 2070s. b Heat map of multivariate environmental similarity surface area in 2070s. Additional File 2: Supplement Figure S6. Multivariate environmental similarity surface and most dissimilar variable analysis under different combinations of climate change scenarios in 2090s. a Multivariate environmental similarity surface and most dissimilar variable in 2090s. b Heat map of multivariate environmental similarity surface area in 2090s. Additional File 2: Supplement Figure S7. Trend surfaces of different climate variables in Shandong and Guizhou provinces (current and SSP585-2090s). Additional File 2: Supplement Figure S8. Interpolation errors and mean square errors of trend surfaces. a Shandong Province (current). b Shandong Province (SSP585-2090s). c Guizhou Province (current). d Guizhou Province (SSP585-2090s). In a-d, the dashed line indicates outliers with a factor of 1, box range represents standard deviation, the solid line in the middle of the box indicates the median, the white squares indicate average, and the grey dots indicate the data for each distribution, the line graphs in gray boxes indicated the corresponding error value of spatial interpolation inversion. Additional File 2: Supplement Figure S9. Clustering analysis of five climate variables. a Current. b SSP585-2090s. The label indicates 151 distribution records, referred to in Table S1; the different colors of the squares indicate the provinces where the distribution records are located.

## Data Availability

The data will be available from the corresponding author upon reasonable request.

## Abbreviations

AUC: Area under the receiver operating characteristic curve
Biomod2: BIOdiversity MODelling2
CMIP: Climate Model Intercomparison Project
CO_2_: Carbon dioxide
CV: Coefficient of variation
GIS: Geographic information system
GRA: Grey relational analysis
MaxEnt: Maximum entropy
PCA: Principal component analysis
ROC: Receiver operating characteristic
S: Similarity
SDM: Species distributed model
SSP: Shared socioeconomic pathway
TSS: Total sum of squares

## References

[CR1] Abdi H, Williams LJ. Principal component analysis. Wiley Interdiscipl Rev Comput Stat. 2010;2(4):433–59.

[CR2] Aitken SN, Sam Y, Jason AH, Wang TL, Sierra CM. Adaptation, migration or extirpation: climate change outcomes for tree populations. Evol Appl. 2008;1(1):95–111. 25567494 10.1111/j.1752-4571.2007.00013.x PMC3352395

[CR3] Akomolafe GF, Zakaria BR. Modelling the distribution of a potential invasive tropical Fern, *Cyclosorus afer* in Nigeria. Afr J Ecol. 2020;58(2):345.

[CR4] Alexander JM, Diez JM, Levine JM. Novel competitors shape species’ responses to climate change. Nature. 2015;525(7570):515–8. 26374998 10.1038/nature14952

[CR5] Barnaud A, Houliston GJ. Population genetics of the threatened tree daisy *Olearia gardneri* (Asteraceae), conservation of a critically endangered species. Conserv Genet. 2010;11(4):1515–22.

[CR6] Broennimann O, Fitzpatrick MC, Pearman PB, Petitpierre B, Pellissier L, Yoccoz NG, Thuiller W, Fortin MJ, Randin C, Zimmermann NE, Graham CH, Guisan A. Measuring ecological niche overlap from occurrence and spatial environmental data. Glob Ecol Biogeogr. 2012;21(4):481–97.

[CR7] Camallec MD, Levin E, David S, Faigenboim A, Foolda MR, Lers A. Molecular and biochemical components associated with chilling tolerance in tomato: comparison of different developmental stages. Mol Hortic. 2024;4(1):31–20. 39232835 10.1186/s43897-024-00108-0 PMC11375913

[CR8] Céline B, Bertelsmeier C, Leadley P, Thuiller W, Courchamp F. Impacts of climate change on the future of biodiversity. Ecol Lett. 2012;15(4):365–77. 22257223 10.1111/j.1461-0248.2011.01736.x PMC3880584

[CR9] Chen IC, Hill JK, Ohlemüller R, Roy DB, Thomas CD. Rapid range shifts of species associated with high levels of climate warming. Science. 2011;333(6045):1024–6. 21852500 10.1126/science.1206432

[CR10] Chen T, Chen Q, Luo Y, Huang ZL, Zhang J, Tang HR, Pan DM, Wang XR. Phylogeography of Chinese cherry (*Prunus pseudocerasus* Lindl.) inferred from chloroplast and nuclear DNA: insights into evolutionary patterns and demographic history. Plant Biol. 2015;17(4):787–97. 25521479 10.1111/plb.12294

[CR11] Chen T, Huang XJ, Zhang J, Chen Q, Liu Y, Tang HR, Pan DM, Wang XR. Genetic diversity and population structure patterns in Chinese cherry (*Prunus pseudocerasus* Lindl) landraces. Plant Mol Biol Rep. 2016;34(2):440–53.

[CR12] Chen T, Wang XR, Tang HR, Chen Q, Huang XJ, Chen J. Genetic diversity and population structure of Chinese cherry revealed by chloroplast DNA TrnQ-rps16 intergenic spacers variation. Genet Resour Crop Ev. 2013;60(6):1859–71.

[CR13] Cui NB, Du TS, Li FS, Tong L, Kang SZ, Wang MX, Liu XZ, Li ZJ. Response of vegetative growth and fruit development to regulated deficit irrigation at different growth stages of pear-jujube tree. Agr Water Manage. 2009;96(8):1237–46.

[CR14] Deng XQ, Xu DP, Liao WK, Wang RL, Zhuo ZH. Predicting the distributions of *Scleroderma guani* (Hymenoptera: Bethylidae) under climate change in China. Ecol Evol. 2022;12(10):E9410. 36225826 10.1002/ece3.9410 PMC9534726

[CR15] Deng XY, Liu Q, Deng Y, Mahadevan S. An improved method to construct basic probability assignment based on the confusion matrix for classification problem. Inform Sci. 2016;340–341:250–61.

[CR16] Di CV, Broennimann V, Petitpierre B, Breiner FT, D’Amen M, Randin C, Engler R, Pottier J, Pio D, Dubuis A, Pellissier L, Mateo RG, Hordijk W, Salamin N, Guisan A. Ecospat: an R package to support spatial analyses and modeling of species niches and distributions. Ecography (Copenhagen). 2017;40(6):774–87.

[CR17] Duan RY, Kong XQ, Huang MY, Varela S, Xiang JX. The potential effects of climate change on amphibian distribution, range fragmentation and turnover in China. PeerJ. 2016;4:E2185. 27547522 10.7717/peerj.2185 PMC4974927

[CR18] Elith J, John L. Species distribution models: ecological explanation and prediction across space and time. Annu Rev Ecol Evol S. 2009;40(1): 677–97.

[CR19] Fadón E, María H, Javier R. Anther and Pollen Development in Sweet Cherry (*Prunus avium* L.) in Relation to Winter Dormancy. Protoplasma. 2019;256(3):733–44. 30506265 10.1007/s00709-018-01332-4

[CR20] Fang W, Duo W, Ge G, Zhang M, Lang J, Wei J. Potential distributions of the invasive barnacle scale *ceroplastes cirripediformis* (Hemiptera: Coccidae) under climate change and implications for its management. J Econ Entomol. 2021;114(1):82–9. 33184624 10.1093/jee/toaa245

[CR21] Guisan A, Petitpierre B, Broennimann O, Daehler C, Kueffer C. Unifying niche shift studies: insights from biological invasions. Trends Eco EvoL (Amsterdam). 2014;29(5):260–9. 10.1016/j.tree.2014.02.009 24656621

[CR22] Guy C, Fatma K, Joachim K, Joachim S, Dirk KH. Metabolomics of temperature stress. Physiol Plantarum. 2008;132(2):220–35. 10.1111/j.1399-3054.2007.00999.x 18251863

[CR23] Hirabayashi K, Murch SJ, Erland LAE. Predicted impacts of climate change on wild and commercial berry habitats will have food security, conservation and agricultural implications. Sci Total Environ. 2022;845:157341. 35842164 10.1016/j.scitotenv.2022.157341

[CR24] Jiu S, Chen B, Dong X, Lv Z, Wang Y, Yin C, Xu Y, Zhang S, Zhu J, Wang J, Liu X, Sun W, Yang G, Li M, Li S, Zhang Z, Liu R, Wang L, Manzoor MA, José Q, Wang S, Lei Y, Yang L, Dirlewanger E, Dong Y, Zhang C. Chromosome-scale genome assembly of *Prunus pusilliflora *provides novel insights into genome evolution, disease resistance, and dormancy release in *Cerasus *L. Hortic Res. 2023;10:uhad062. 10.1093/hr/uhad062 PMC10200261 37220556

[CR25] Jiu S, Lv Z, Liu M, X u Y, Chen B, Dong X, Zhang X, Gao J, Manzoor MA, Xia M, Li F, Li H, Chen L, Zhang X, Wang S, Dong Y, Zhang C. Haplotype-resolved genome assembly for tetraploid Chinese Cherry (*Prunus pseudocerasus*) offers insights into fruit firmness. Hortic Res. 2024b;7:uhae142. 10.1093/hr/uhae142 PMC11233885 38988622

[CR26] Jiu S, Manzoor MA, Chen B, Xu Y, Abdullah M, Zhang X, Lv Z, Zhu J, Cao J, Liu X, Wang J, Liu R, Wang S, Dong Y, Zhang C. Chromosome-level genome assembly provides insights into genetic diversity, evolution, and flower development of *Prunus conradinae*. Mol Hortic. 2024a;4:25. 10.1186/s43897-024-00101-7 PMC11186256 38898491

[CR27] Jordi LP, Zhang FM, Sun HQ, Ying TS, Song G. Centres of plant endemism in China: places for survival or for speciation? J Biogeogr. 2011;38(7):1267–80.

[CR28] Kristine G, Rodel L, Amor I, Bradfield L, Florencia P. Predicting geographic distribution and habitat suitability due to climate change of selected threatened forest tree species in the Philippines. Appl Geogr. 2013;44:12–22.

[CR29] Kumar S, Lisa GN, Wee LY. Assessing the potential for establishment of western cherry fruit fly using ecological niche modeling. J Econ Entomol. 2014;107(3):1032–44. 25026662 10.1603/ec14052

[CR30] Lake TA, Runquist RBD, Moeller DA, Bellard C. Predicting range expansion of invasive species: pitfalls and best practices for obtaining biologically realistic projections. Divers Distrib. 2020;26(12):1767–79.

[CR31] Lasky JR, Bachelot B, Muscarella R, Schwartz N, Forero-Montaa J, Nytch CJ, Swenson NG, Thompson J, Zimmerman JK, Uriarte M. Ontogenetic shifts in trait-mediated mechanisms of plant community assembly. Ecology. 2015;96:2157. 26405741 10.1890/14-1809.1

[CR32] Li B, Xie Z, Zhang A, Xu W, Zhang C, Liu Q, Liu C, Wang S. Tree growth characteristics and flower bud differentiation of sweet cherry (*Prunus avium* L.) under different climate conditions in China. Hortic Sci. 2010a;37(1):6–13.

[CR33] Li JJ, Fan G, He Y. Predicting the current and future distribution of three *Coptis* herbs in China under climate change conditions, using the MaxEnt model and chemical analysis. Sci Total Environ. 2020;698:e134141. 10.1016/j.scitotenv.2019.134141 31505366

[CR34] Li YQ, Chen WR, Xin DY, Li ZJ, Guo WD. Effects of temperature on flower bud germination of *Prunus pseudocerasus*. J Anhui Agri Sci. 2010b;38(31):17604–8+17631. (in chinese and english abstract)

[CR35] Liu Y, Huang P, Lin FR, Yang WY, Hannes G, Kettle C, Zheng YQ. MaxEnt modelling for predicting the potential distribution of a near threatened rosewood species (*Dalbergia cultrata* Graham Ex Benth). Ecol Eng. 2019;141:105612.

[CR36] Lu HY, Wu WH, Jarukitt L, Yang DM, Xiao GN, Luo ZS, Li L. Effect of micro-perforated film packing on fatty acid-derived volatile metabolism of “Red Globe” table grapes. Food Bioprocess Tech. 2018;11(10):1807–17.

[CR37] Mitchell JFB, Johns TC, Ingram WJ, Lowe JA. The effect of stabilising atmospheric carbon dioxide concentrations on global and regional climate change. Geophys Res Lett. 2000;27(18):2977–80.

[CR38] Nadine B, Christoph S, Willy T. Impact of holocene climate changes on alpine and treeline vegetation at sanetsch pass, Bernese Alps, Switzerland. Rev Palaeobot Palyno. 2012;174:91–100.

[CR39] Olsen JE. Light and temperature sensing and signaling in induction of bud dormancy in woody plants. Plant Mol Biol. 2010;73(1–2):37–47. 10.1007/s11103-010-9620-9 20213333

[CR40] Paltineanu C, Emil C. Climate change impact on phenological stages of sweet and sour cherry trees in a continental climate environment. Sci Hortic. 2020;261: 109011.

[CR41] Parmesan C. Climate and species’ range. Nature. 1996;382(6594):765–6.

[CR42] Phillips SJ, Anderson RP, Schapire RE. Maximum entropy modeling of species geographic distributions. Ecol Model. 2006;190(3/4):231–59.

[CR43] Primack RB, Hiroyoshi H, Abraham JMR. The impact of climate change on cherry trees and other species in Japan. Biol Conserv. 2009;142(9):1943–9.

[CR44] Rivera-Parra JL, Peña-Loyola PJ. Potential high-quality growing tea regions in ecuador: an alternative cash crop for ecuadorian small landholders. J Sci Food Agr. 2020;100(4):1827–31. 31875429 10.1002/jsfa.10225

[CR45] Rumpf SB, Karl H, Johannes W, Wolfgang W, Dietmar M, Andreas G, Günther K, Niklaus EZ, Stefan D. Extinction debts and colonization credits of non-forest plants in the european alps. Nat Commun. 2019;10(1):4293–9. 31541105 10.1038/s41467-019-12343-x PMC6754411

[CR46] Saranya KRL, Lakshmi TV, Reddy CS. Predicting the potential sites of *Chromolaena Odorata* and *Lantana Camara* in forest landscape of eastern ghats using habitat suitability models. Ecol Inform. 2021;66:101455.

[CR47] Searcy CA, Shaffer HB, Ackerly DD, Judith LB. Do ecological niche models accurately identify climatic determinants of species ranges? AM NAT. 2016;187(4):423–35. 27028071 10.1086/685387

[CR48] Segurado P, Araújo MB. An evaluation of methods for modelling species distributions. J Biogeogr. 2004;31(10):1555–68.

[CR49] Sharifian S, Mohammad SM, Seyedeh LMN. Modeling present distribution commercial fish and shrimps using MaxEnt. Wetlands. 2021;42(5): e39.

[CR50] Shi PJ, Sun S, Wang M, Li N, Wang JA, Jin YY, Gu XT. Climate change regionalization in China (1961–2010). Sci China Earth Sci. 2014;57(011):2676–89.

[CR51] Suran P, Vávra R, Jonáš M, Zelený L, Skřivanová A. Effect of rain protective covering of sweet cherry orchard on fruit quality and cracking. Acta Hortic. 2019;1235:189–96.

[CR52] Swets J. Measuring the accuracy of diagnostic systems. Science. 1988;240(4857):1285–93. 3287615 10.1126/science.3287615

[CR53] Thuiller W. Climate change and the ecologist. Nature. 2007;448(7153):550–2. 17671497 10.1038/448550a

[CR54] Thuiller W, Lavorel S, Araujo MB, Sykes MT, Prentice IC. Climate change threats to plant diversity in Europe. PNAS. 2005;102(23):8245–50. 15919825 10.1073/pnas.0409902102 PMC1140480

[CR55] Thuiller W, Lavergne S, Roquet C, Boulangeat I, Lafourcade B, Araujo MB. Consequences of climate change on the tree of life in Europe. Nature. 2011;470(7335):531–4. 21326204 10.1038/nature09705

[CR56] Wan J, Wang C, Han S. Planning the priority protected areas of endangered orchid species in Northeastern China. Biodivers Conserv. 2014;23(6):1395–409.

[CR57] Wan JZ, Wang CJ, Liu CX, Li HL. Climate change may alter genetic diversity of *Duchesnea indica*, a clonal plant species. Biochem Syst Ecol. 2016;66:114–22.

[CR58] Wang PJ, Chen XJ, Guo YC, Zheng YC, Yue C, Yang JF, Ye NX. Identification of CBF transcription factors in tea plants and a survey of potential CBF target genes under low temperature. Int J Mol Sci. 2019;20(20):5137. 31627263 10.3390/ijms20205137 PMC6829267

[CR59] Wang Y, Hu GP, Liu ZS, Zhang J, Ma L, Tian T, Wang H, Chen T, Chen Q, He W, Yang SF, Lin YX, Zhang YT, Li MY, Zhang Y, Luo Y, Tang HR, Wang XR. Phenotyping in flower and main fruit traits of Chinese Cherry [*Cerasus pseudocerasus* (Lindl.) G.Don]. Sci Hortic. 2022;296:110920.

[CR60] Wang Y, Xu Y, Xu J, Sun W, Lv Z, Manzoor MA, Liu X, Shen Z, Wang J, Liu R, Whiting MD, Jiu S, Zhang C. Oxygenation alleviates waterlogging-caused damages to cherry rootstocks. Mol Hortic. 2023;3:8. 37789432 10.1186/s43897-023-00056-1 PMC10515082

[CR61] Woodward FI, Williams BG. Climate and plant distribution at global and local scales. Vegetatio. 1987;69:189–97.

[CR62] Wu YD, Yu XK, Suo N, Bai HQ, Ke QZ, Chen J, Pan Y, Zheng WQ, Xu P. Thermal tolerance, safety margins and acclimation capacity assessments reveal the climate vulnerability of large yellow croaker aquaculture. Aquaculture. 2022;561:738665.

[CR63] Wu Z, Liang JH, Li T, Zhang DH, Teng NJ. A LlMYB305-LlC3H18-LlWRKY33 module regulates thermotolerance in Lily. Mol Hortic. 2023;3(1):15. 37789438 10.1186/s43897-023-00064-1 PMC10514960

[CR64] Wu ZY, Chen XQ, et al. Flora of China, vol. 9. Beijing: Science Press & St. Louis: Missouri Botanical Garden Press; 2003.

[CR65] Xian XQ, Zhao HX, Wang R, Huang HK, Chen BX, Zhang GF, Liu WX, Wan FH. Climate change has increased the global threats posed by three ragweeds (*Ambrosia* L.) in the Anthropocene. Sci Total Environ. 2023;859: 160252. 36427731 10.1016/j.scitotenv.2022.160252

[CR66] Xie T, Liu RH, Wei ZY. Improvement of the fast clustering algorithm improved by K-means in the big data. Appl Math Nonlinear Sci. 2020;5(1):1–10.

[CR67] Xu DP, Zhuo ZH, Wang RL, Ye M, Pu B. Modeling the distribution of *Zanthoxylum armatum* in China with MaxEnt modeling. Glob Ecol Conserv. 2019;19: e00691.

[CR68] Xu N, Meng FY, Zhou GF, Li YF, Wang B, Lu H. Assessing the suitable cultivation areas for *Scutellaria baicalensis* in China using the MaxEnt model and multiple linear regression. Biochem Syst Ecol. 2020;90:104052.

[CR69] Yan DT, Chen W, Liu L, Li J, Liu L, Wang YL. Change in current and future geographic distributions of *Ulmus lamellosa* in China. J Forestry Res. 2017;29(4):1147–56.

[CR70] Yan HY, Feng L, Zhao YF, Feng L, Wu D, Zhu CP. Prediction of the spatial distribution of *Alternanthera philoxeroides* in China based on ArcGIS and MaxEnt. Glob Ecol Conserv. 2020;21:E00856.

[CR71] Yan HY, He J, Xu XC, Yao XY, Wang GY, Tang LG, Lei F, Zou LM, Gu XL, Qu YF, Qu LF. Prediction of potentially suitable distributions of *Codonopsis pilosula* in China based on an optimized MaxEnt model. Front Ecol Evol. 2021;9:773396.

[CR72] Yang Y, Wu ZF, Guo L, He HS, Ling YH, Wang L, Zong SW, Na RS, Du HB, Li MH. Effects of winter chilling vs. spring forcing on the spring phenology of trees in a cold region and a warmer reference region. Sci Total Environ. 2020;725:138323. 32298892 10.1016/j.scitotenv.2020.138323

[CR73] Zhang J, Tao C, Wang Y, Chen Q, Sun B, Luo Y, Zhang Y, Tang HR, Wang XR. Genetic diversity and domestication footprints of Chinese Cherry [*Cerasus pseudocerasus* (Lindl.) G.Don] as revealed by nuclear microsatellites. Front Plant Sci. 2018;9:238. 29535750 10.3389/fpls.2018.00238 PMC5835088

[CR74] Zhang X, Srinivasan R. Gis-based spatial precipitation estimation using next generation radar and raingauge data. Environ Modell Softw. 2010;25(12):1781–8.

[CR75] Zhao WL, Mao QG, Liu GL, Li YQ, Xia JB, Zhang YJ. Patterns of compound-leaf form and deciduous-leaf habit across forests in China: their association and key climatic factors. Sci Total Environ. 2022;851:158108. 35987224 10.1016/j.scitotenv.2022.158108

[CR76] Zhao Y, Cao H, Xu W, Chen G, Lian J, Du Y, Ma K. Contributions of precipitation and temperature to the large scale geographic distribution of fleshy-fruited plant species: Growth form matters. Sci Rep. 2018;8(1):17017. 30451937 10.1038/s41598-018-35436-x PMC6243012

[CR77] Zhou JW, Li QQ. Stress responses of plants through transcriptome plasticity by mRNA alternative polyadenylation. Mol Hortic. 2023;3(1):19. 37789388 10.1186/s43897-023-00066-z PMC10536700

[CR78] Zhu H, You LX, Li YF, Wang HC, Wang XR. Modeling the geographical distribution pattern and climatic limited factors of *Cerasus schneideriana*. J Trop Subtrop Bot. 2017;25(04):315–22 (in chinese and english abstract).

[CR79] Zhu SX, Zhu H, Cheng L, Yi XG, Wang XR. Modeling geographical distribution pattern and comparison of ecological characteristics between *Cerasus cerasoides* and *C. campanulata*. Guihaia. 2019;39(10):1398–406 (in chinese and english abstract).

