# Supplementary figures and images for: Climate change affects the suitability of Chinese cherry (Prunus pseudocerasus Lindl.) in China
Source: Mol Hortic. 2025 Mar 6;5:26. doi: 10.1186/s43897-024-00136-w (PMC11884058; doi:10.1186/s43897-024-00136-w)

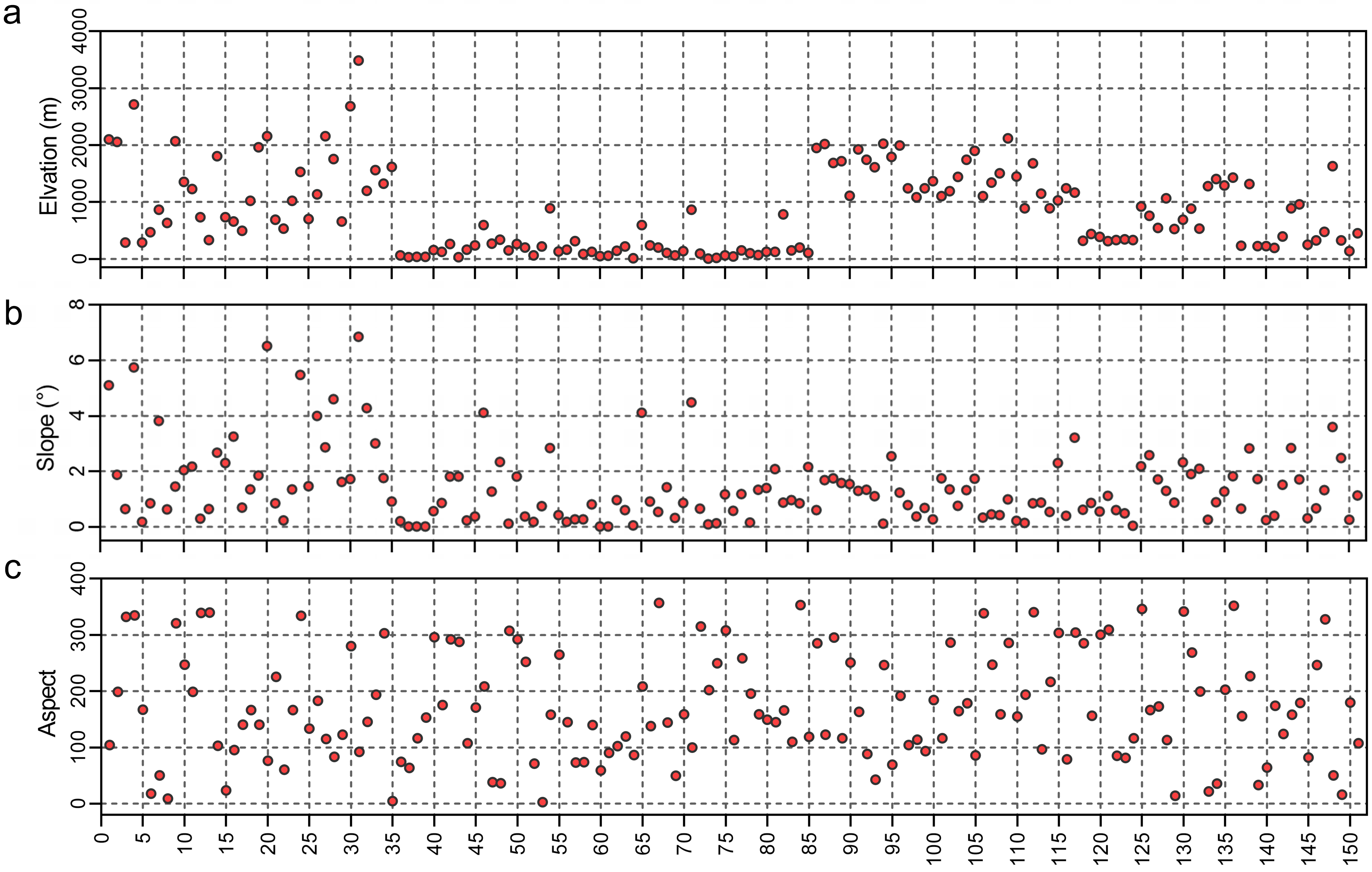

Supplement: Supplementary file 2 — Additional File 2: Supplement Figure S1. The landform with 151 distribution records of P. pseudocerasus. a Elevation. b Slope. c Aspect. Additional File 2: Supplement Figure S2. Ecological niche comparisons of P. pseudocerasus under different pathways in 2050s. a Ecological niches of P. pseudocerasus with SSP126-2050s–SSP585-2050s. Red arrows indicate Schoener's D. Blue indicates ecological niche overlap, green indicates unfilling, and red indicates expansion. b Alternative verification method of ecological niches with SSP126-2050s–SSP585-2050s. c Ecological niche similarity of different pathways in 2050s. The red arrow indicates the centroids of each species' realized ecological niche. Additional File 2: Supplement Figure S3. Ecological niche comparisons of P. pseudocerasus under different pathways in 2070s. a Ecological niches of P. pseudocerasus with SSP126-2070s–SSP585-2070s. Red arrows indicate Schoener's D. Blue indicates ecological niche overlap, green indicates unfilling, and red indicates expansion. b Alternative verification method of ecological niches with SSP126-2070s–SSP585-2070s. c Ecological niche similarity of different pathways in 2070s. The red arrow indicates the centroids of each species' realized ecological niche. Additional File 2: Supplement Figure S4. Multivariate environmental similarity surface and most dissimilar variable analysis under different combinations of climate change scenarios in 2050s. a Multivariate environmental similarity surface and most dissimilar variable in 2050s. b Heat map of multivariate environmental similarity surface area in 2050s. Additional File 2: Supplement Figure S5. Multivariate environmental similarity surface and most dissimilar variable analysis under different combinations of climate change scenarios in 2070s. a Multivariate environmental similarity surface and most dissimilar variable in 2070s. b Heat map of multivariate environmental similarity surface area in 2070s. Additional File 2: Supplement Figure [file 43897_2024_136_MOESM2_ESM.zip › Supplementary Figures/Fig.S1.tif]

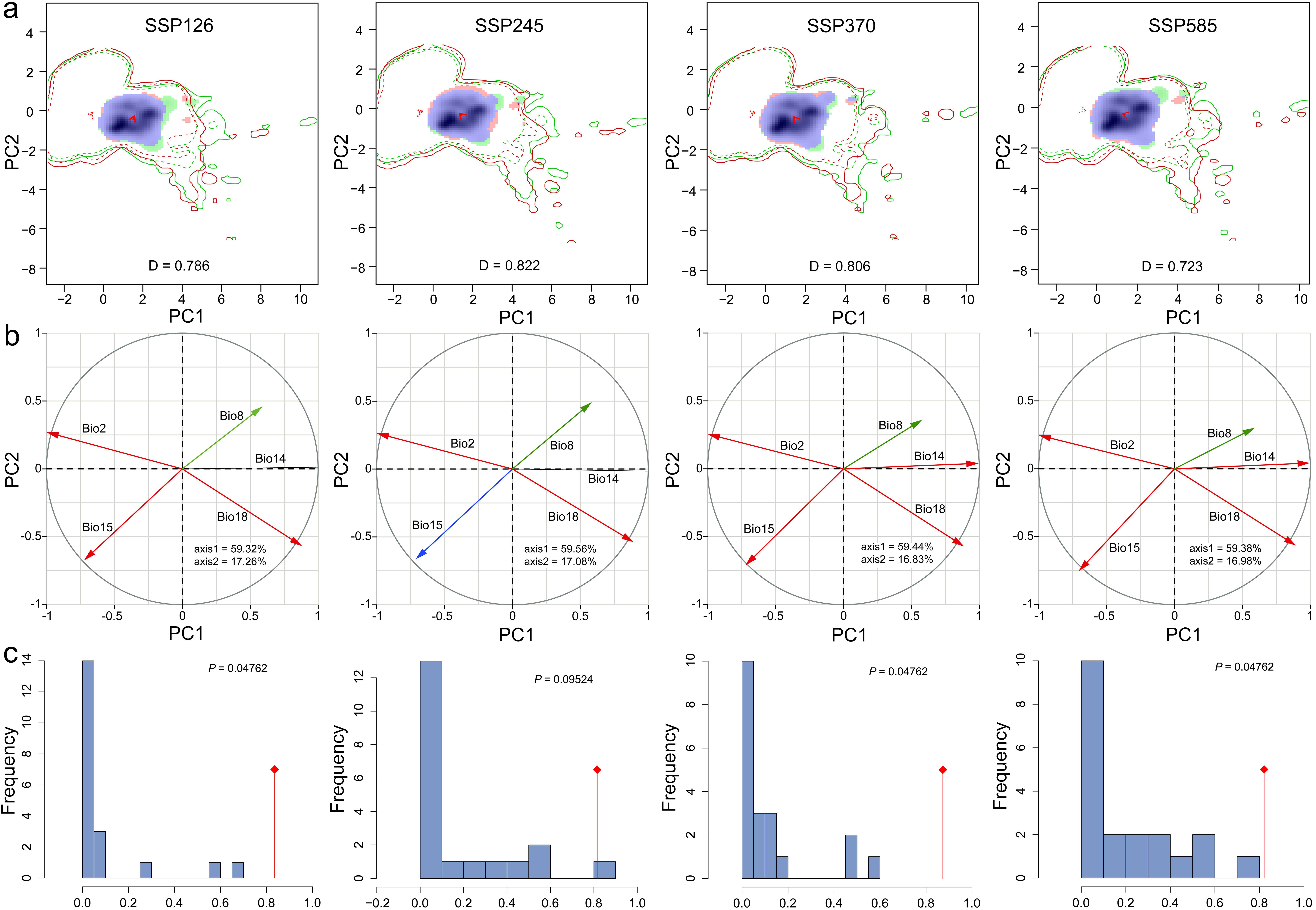

Supplement: Supplementary file 2 — Additional File 2: Supplement Figure S1. The landform with 151 distribution records of P. pseudocerasus. a Elevation. b Slope. c Aspect. Additional File 2: Supplement Figure S2. Ecological niche comparisons of P. pseudocerasus under different pathways in 2050s. a Ecological niches of P. pseudocerasus with SSP126-2050s–SSP585-2050s. Red arrows indicate Schoener's D. Blue indicates ecological niche overlap, green indicates unfilling, and red indicates expansion. b Alternative verification method of ecological niches with SSP126-2050s–SSP585-2050s. c Ecological niche similarity of different pathways in 2050s. The red arrow indicates the centroids of each species' realized ecological niche. Additional File 2: Supplement Figure S3. Ecological niche comparisons of P. pseudocerasus under different pathways in 2070s. a Ecological niches of P. pseudocerasus with SSP126-2070s–SSP585-2070s. Red arrows indicate Schoener's D. Blue indicates ecological niche overlap, green indicates unfilling, and red indicates expansion. b Alternative verification method of ecological niches with SSP126-2070s–SSP585-2070s. c Ecological niche similarity of different pathways in 2070s. The red arrow indicates the centroids of each species' realized ecological niche. Additional File 2: Supplement Figure S4. Multivariate environmental similarity surface and most dissimilar variable analysis under different combinations of climate change scenarios in 2050s. a Multivariate environmental similarity surface and most dissimilar variable in 2050s. b Heat map of multivariate environmental similarity surface area in 2050s. Additional File 2: Supplement Figure S5. Multivariate environmental similarity surface and most dissimilar variable analysis under different combinations of climate change scenarios in 2070s. a Multivariate environmental similarity surface and most dissimilar variable in 2070s. b Heat map of multivariate environmental similarity surface area in 2070s. Additional File 2: Supplement Figure [file 43897_2024_136_MOESM2_ESM.zip › Supplementary Figures/Fig.S2.tif]

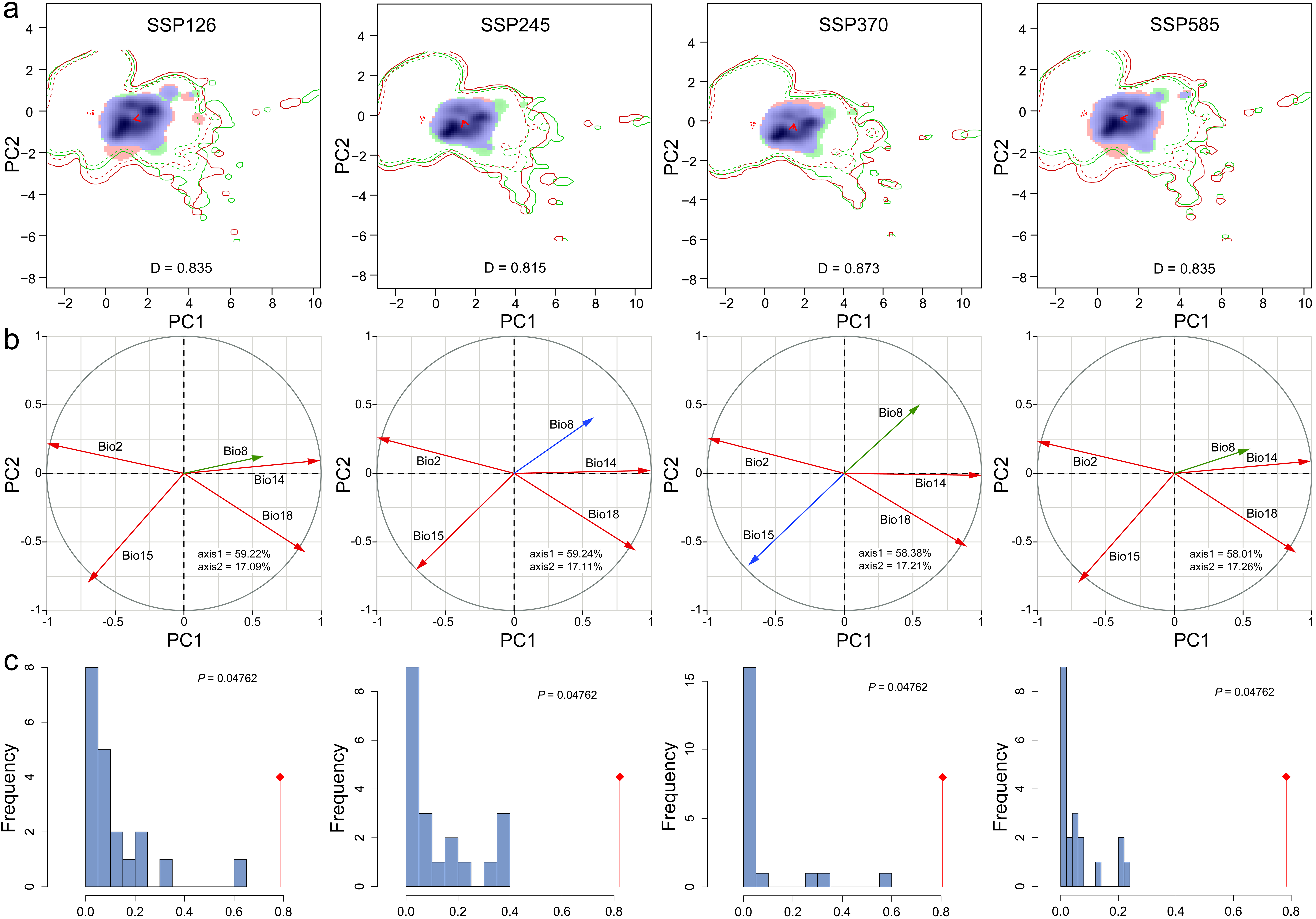

Supplement: Supplementary file 2 — Additional File 2: Supplement Figure S1. The landform with 151 distribution records of P. pseudocerasus. a Elevation. b Slope. c Aspect. Additional File 2: Supplement Figure S2. Ecological niche comparisons of P. pseudocerasus under different pathways in 2050s. a Ecological niches of P. pseudocerasus with SSP126-2050s–SSP585-2050s. Red arrows indicate Schoener's D. Blue indicates ecological niche overlap, green indicates unfilling, and red indicates expansion. b Alternative verification method of ecological niches with SSP126-2050s–SSP585-2050s. c Ecological niche similarity of different pathways in 2050s. The red arrow indicates the centroids of each species' realized ecological niche. Additional File 2: Supplement Figure S3. Ecological niche comparisons of P. pseudocerasus under different pathways in 2070s. a Ecological niches of P. pseudocerasus with SSP126-2070s–SSP585-2070s. Red arrows indicate Schoener's D. Blue indicates ecological niche overlap, green indicates unfilling, and red indicates expansion. b Alternative verification method of ecological niches with SSP126-2070s–SSP585-2070s. c Ecological niche similarity of different pathways in 2070s. The red arrow indicates the centroids of each species' realized ecological niche. Additional File 2: Supplement Figure S4. Multivariate environmental similarity surface and most dissimilar variable analysis under different combinations of climate change scenarios in 2050s. a Multivariate environmental similarity surface and most dissimilar variable in 2050s. b Heat map of multivariate environmental similarity surface area in 2050s. Additional File 2: Supplement Figure S5. Multivariate environmental similarity surface and most dissimilar variable analysis under different combinations of climate change scenarios in 2070s. a Multivariate environmental similarity surface and most dissimilar variable in 2070s. b Heat map of multivariate environmental similarity surface area in 2070s. Additional File 2: Supplement Figure [file 43897_2024_136_MOESM2_ESM.zip › Supplementary Figures/Fig.S3.tif]

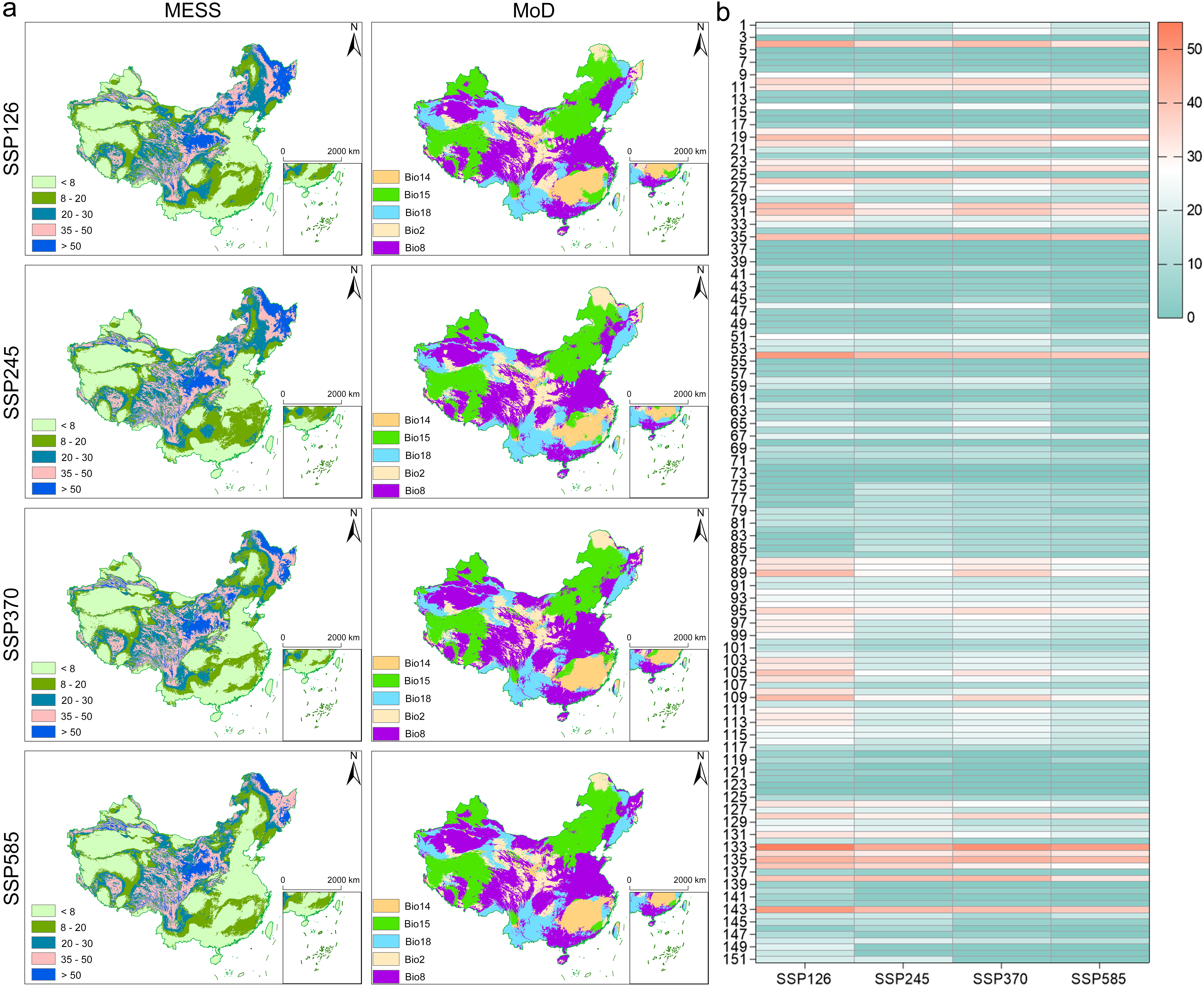

Supplement: Supplementary file 2 — Additional File 2: Supplement Figure S1. The landform with 151 distribution records of P. pseudocerasus. a Elevation. b Slope. c Aspect. Additional File 2: Supplement Figure S2. Ecological niche comparisons of P. pseudocerasus under different pathways in 2050s. a Ecological niches of P. pseudocerasus with SSP126-2050s–SSP585-2050s. Red arrows indicate Schoener's D. Blue indicates ecological niche overlap, green indicates unfilling, and red indicates expansion. b Alternative verification method of ecological niches with SSP126-2050s–SSP585-2050s. c Ecological niche similarity of different pathways in 2050s. The red arrow indicates the centroids of each species' realized ecological niche. Additional File 2: Supplement Figure S3. Ecological niche comparisons of P. pseudocerasus under different pathways in 2070s. a Ecological niches of P. pseudocerasus with SSP126-2070s–SSP585-2070s. Red arrows indicate Schoener's D. Blue indicates ecological niche overlap, green indicates unfilling, and red indicates expansion. b Alternative verification method of ecological niches with SSP126-2070s–SSP585-2070s. c Ecological niche similarity of different pathways in 2070s. The red arrow indicates the centroids of each species' realized ecological niche. Additional File 2: Supplement Figure S4. Multivariate environmental similarity surface and most dissimilar variable analysis under different combinations of climate change scenarios in 2050s. a Multivariate environmental similarity surface and most dissimilar variable in 2050s. b Heat map of multivariate environmental similarity surface area in 2050s. Additional File 2: Supplement Figure S5. Multivariate environmental similarity surface and most dissimilar variable analysis under different combinations of climate change scenarios in 2070s. a Multivariate environmental similarity surface and most dissimilar variable in 2070s. b Heat map of multivariate environmental similarity surface area in 2070s. Additional File 2: Supplement Figure [file 43897_2024_136_MOESM2_ESM.zip › Supplementary Figures/Fig.S4.tif]

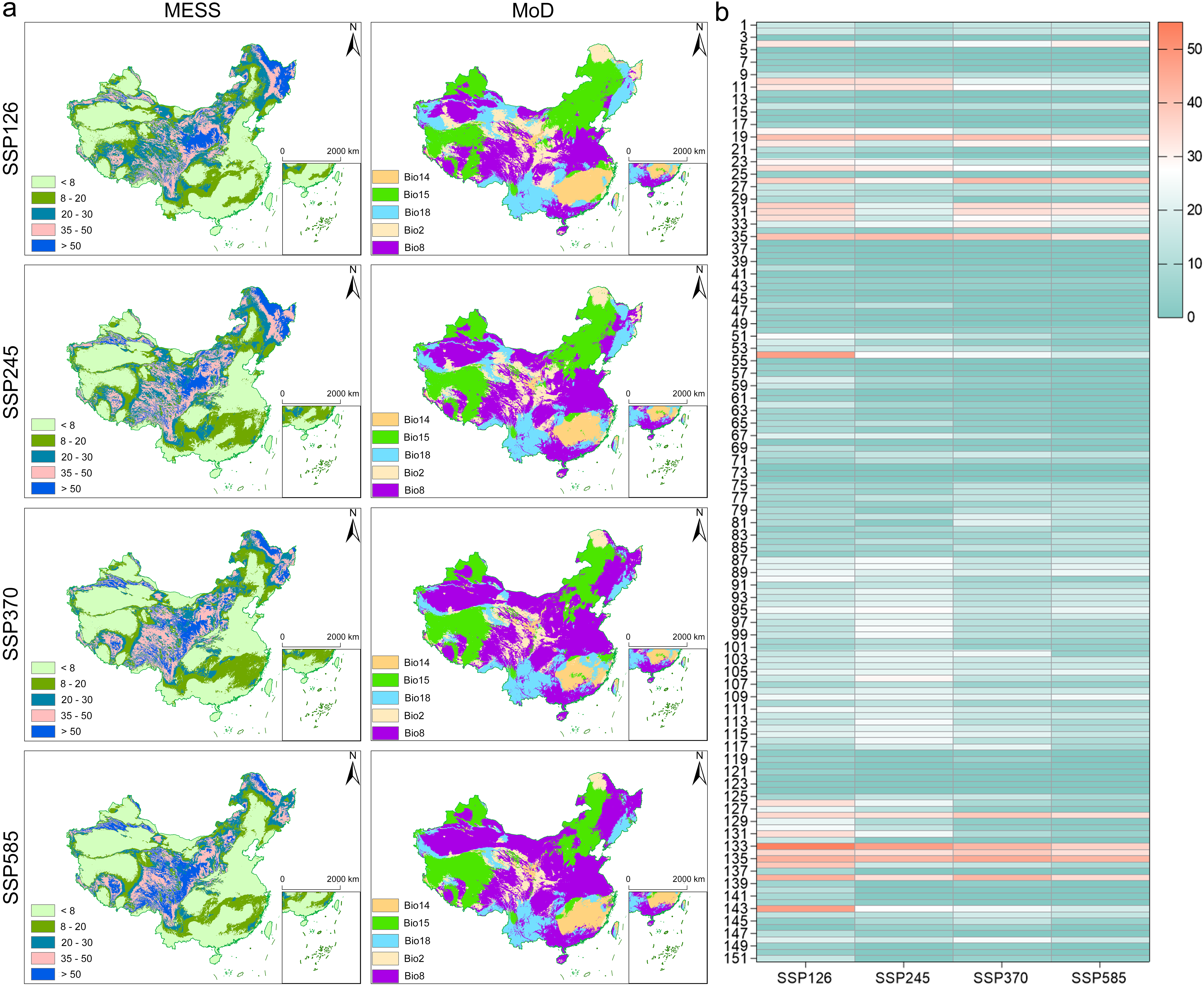

Supplement: Supplementary file 2 — Additional File 2: Supplement Figure S1. The landform with 151 distribution records of P. pseudocerasus. a Elevation. b Slope. c Aspect. Additional File 2: Supplement Figure S2. Ecological niche comparisons of P. pseudocerasus under different pathways in 2050s. a Ecological niches of P. pseudocerasus with SSP126-2050s–SSP585-2050s. Red arrows indicate Schoener's D. Blue indicates ecological niche overlap, green indicates unfilling, and red indicates expansion. b Alternative verification method of ecological niches with SSP126-2050s–SSP585-2050s. c Ecological niche similarity of different pathways in 2050s. The red arrow indicates the centroids of each species' realized ecological niche. Additional File 2: Supplement Figure S3. Ecological niche comparisons of P. pseudocerasus under different pathways in 2070s. a Ecological niches of P. pseudocerasus with SSP126-2070s–SSP585-2070s. Red arrows indicate Schoener's D. Blue indicates ecological niche overlap, green indicates unfilling, and red indicates expansion. b Alternative verification method of ecological niches with SSP126-2070s–SSP585-2070s. c Ecological niche similarity of different pathways in 2070s. The red arrow indicates the centroids of each species' realized ecological niche. Additional File 2: Supplement Figure S4. Multivariate environmental similarity surface and most dissimilar variable analysis under different combinations of climate change scenarios in 2050s. a Multivariate environmental similarity surface and most dissimilar variable in 2050s. b Heat map of multivariate environmental similarity surface area in 2050s. Additional File 2: Supplement Figure S5. Multivariate environmental similarity surface and most dissimilar variable analysis under different combinations of climate change scenarios in 2070s. a Multivariate environmental similarity surface and most dissimilar variable in 2070s. b Heat map of multivariate environmental similarity surface area in 2070s. Additional File 2: Supplement Figure [file 43897_2024_136_MOESM2_ESM.zip › Supplementary Figures/Fig.S5.tif]

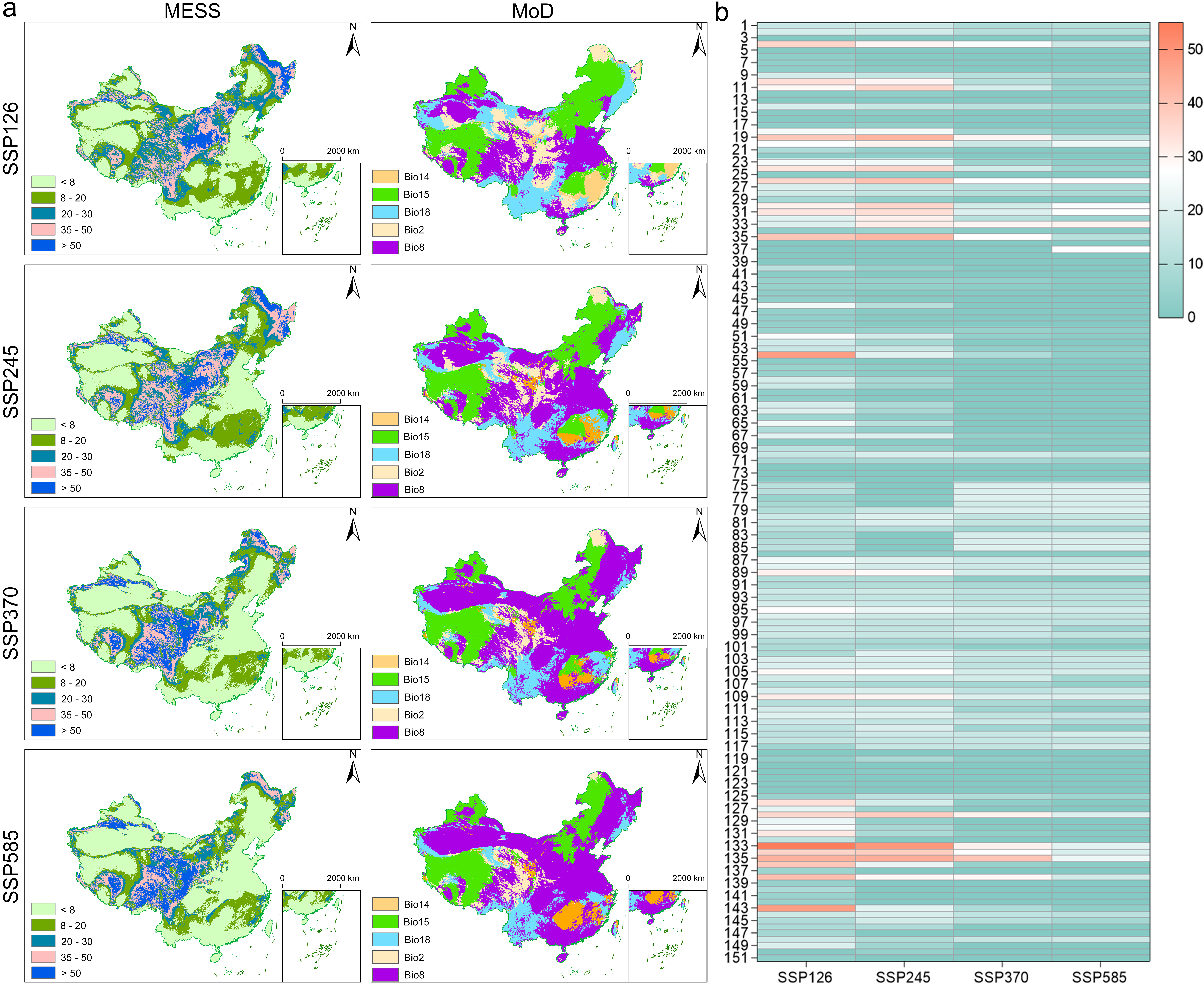

Supplement: Supplementary file 2 — Additional File 2: Supplement Figure S1. The landform with 151 distribution records of P. pseudocerasus. a Elevation. b Slope. c Aspect. Additional File 2: Supplement Figure S2. Ecological niche comparisons of P. pseudocerasus under different pathways in 2050s. a Ecological niches of P. pseudocerasus with SSP126-2050s–SSP585-2050s. Red arrows indicate Schoener's D. Blue indicates ecological niche overlap, green indicates unfilling, and red indicates expansion. b Alternative verification method of ecological niches with SSP126-2050s–SSP585-2050s. c Ecological niche similarity of different pathways in 2050s. The red arrow indicates the centroids of each species' realized ecological niche. Additional File 2: Supplement Figure S3. Ecological niche comparisons of P. pseudocerasus under different pathways in 2070s. a Ecological niches of P. pseudocerasus with SSP126-2070s–SSP585-2070s. Red arrows indicate Schoener's D. Blue indicates ecological niche overlap, green indicates unfilling, and red indicates expansion. b Alternative verification method of ecological niches with SSP126-2070s–SSP585-2070s. c Ecological niche similarity of different pathways in 2070s. The red arrow indicates the centroids of each species' realized ecological niche. Additional File 2: Supplement Figure S4. Multivariate environmental similarity surface and most dissimilar variable analysis under different combinations of climate change scenarios in 2050s. a Multivariate environmental similarity surface and most dissimilar variable in 2050s. b Heat map of multivariate environmental similarity surface area in 2050s. Additional File 2: Supplement Figure S5. Multivariate environmental similarity surface and most dissimilar variable analysis under different combinations of climate change scenarios in 2070s. a Multivariate environmental similarity surface and most dissimilar variable in 2070s. b Heat map of multivariate environmental similarity surface area in 2070s. Additional File 2: Supplement Figure [file 43897_2024_136_MOESM2_ESM.zip › Supplementary Figures/Fig.S6.tif]

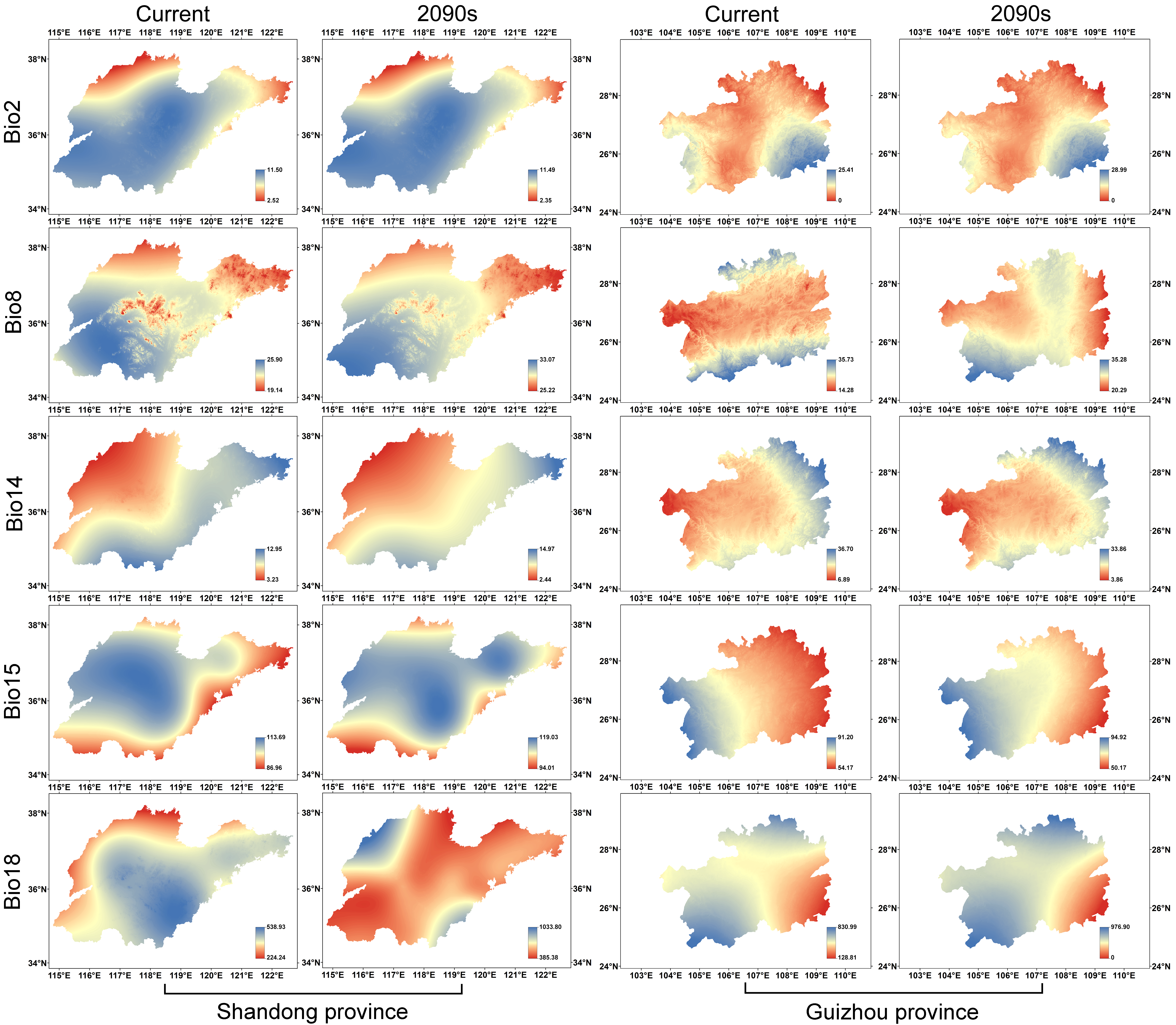

Supplement: Supplementary file 2 — Additional File 2: Supplement Figure S1. The landform with 151 distribution records of P. pseudocerasus. a Elevation. b Slope. c Aspect. Additional File 2: Supplement Figure S2. Ecological niche comparisons of P. pseudocerasus under different pathways in 2050s. a Ecological niches of P. pseudocerasus with SSP126-2050s–SSP585-2050s. Red arrows indicate Schoener's D. Blue indicates ecological niche overlap, green indicates unfilling, and red indicates expansion. b Alternative verification method of ecological niches with SSP126-2050s–SSP585-2050s. c Ecological niche similarity of different pathways in 2050s. The red arrow indicates the centroids of each species' realized ecological niche. Additional File 2: Supplement Figure S3. Ecological niche comparisons of P. pseudocerasus under different pathways in 2070s. a Ecological niches of P. pseudocerasus with SSP126-2070s–SSP585-2070s. Red arrows indicate Schoener's D. Blue indicates ecological niche overlap, green indicates unfilling, and red indicates expansion. b Alternative verification method of ecological niches with SSP126-2070s–SSP585-2070s. c Ecological niche similarity of different pathways in 2070s. The red arrow indicates the centroids of each species' realized ecological niche. Additional File 2: Supplement Figure S4. Multivariate environmental similarity surface and most dissimilar variable analysis under different combinations of climate change scenarios in 2050s. a Multivariate environmental similarity surface and most dissimilar variable in 2050s. b Heat map of multivariate environmental similarity surface area in 2050s. Additional File 2: Supplement Figure S5. Multivariate environmental similarity surface and most dissimilar variable analysis under different combinations of climate change scenarios in 2070s. a Multivariate environmental similarity surface and most dissimilar variable in 2070s. b Heat map of multivariate environmental similarity surface area in 2070s. Additional File 2: Supplement Figure [file 43897_2024_136_MOESM2_ESM.zip › Supplementary Figures/Fig.S7.tif]

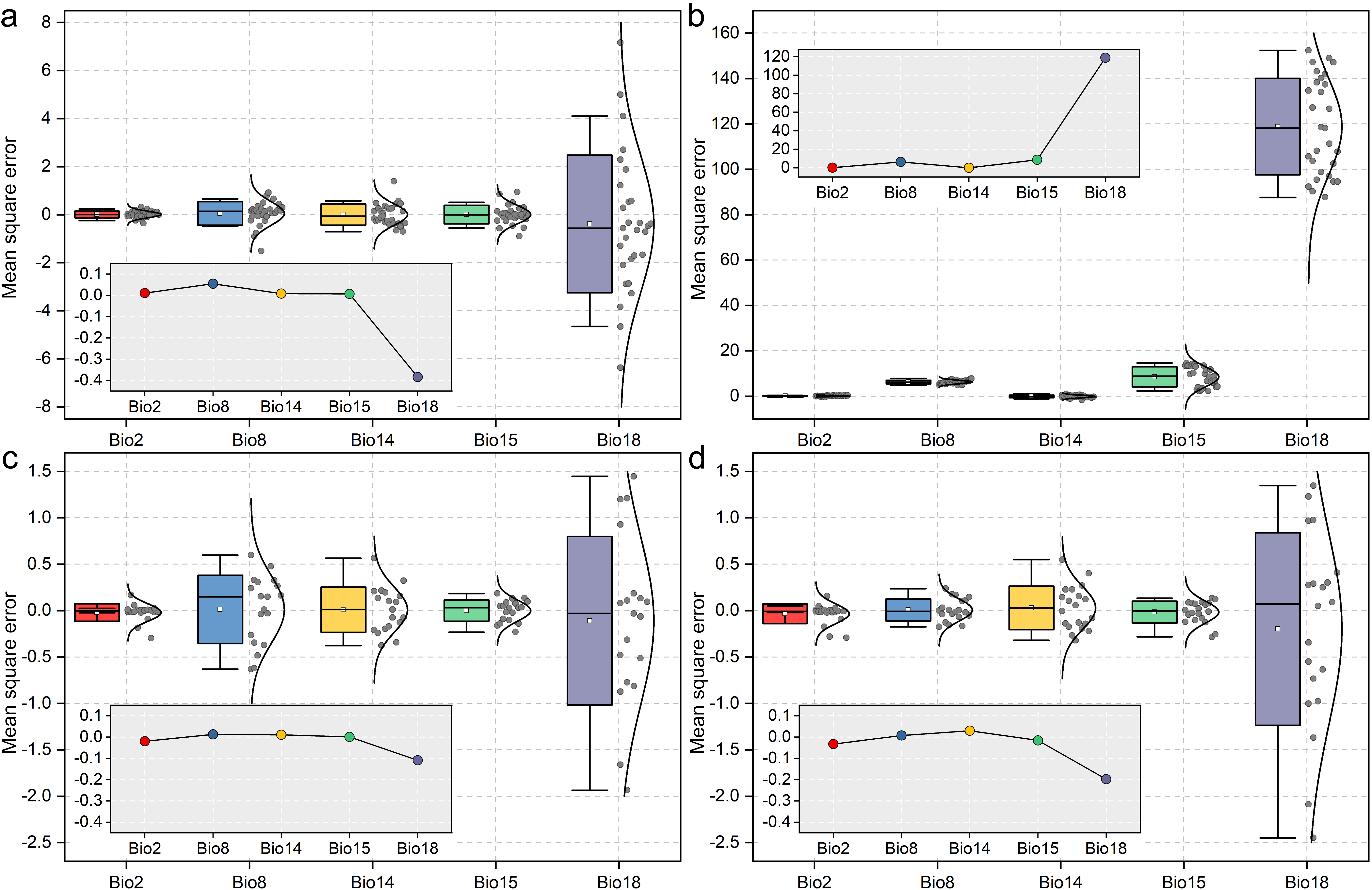

Supplement: Supplementary file 2 — Additional File 2: Supplement Figure S1. The landform with 151 distribution records of P. pseudocerasus. a Elevation. b Slope. c Aspect. Additional File 2: Supplement Figure S2. Ecological niche comparisons of P. pseudocerasus under different pathways in 2050s. a Ecological niches of P. pseudocerasus with SSP126-2050s–SSP585-2050s. Red arrows indicate Schoener's D. Blue indicates ecological niche overlap, green indicates unfilling, and red indicates expansion. b Alternative verification method of ecological niches with SSP126-2050s–SSP585-2050s. c Ecological niche similarity of different pathways in 2050s. The red arrow indicates the centroids of each species' realized ecological niche. Additional File 2: Supplement Figure S3. Ecological niche comparisons of P. pseudocerasus under different pathways in 2070s. a Ecological niches of P. pseudocerasus with SSP126-2070s–SSP585-2070s. Red arrows indicate Schoener's D. Blue indicates ecological niche overlap, green indicates unfilling, and red indicates expansion. b Alternative verification method of ecological niches with SSP126-2070s–SSP585-2070s. c Ecological niche similarity of different pathways in 2070s. The red arrow indicates the centroids of each species' realized ecological niche. Additional File 2: Supplement Figure S4. Multivariate environmental similarity surface and most dissimilar variable analysis under different combinations of climate change scenarios in 2050s. a Multivariate environmental similarity surface and most dissimilar variable in 2050s. b Heat map of multivariate environmental similarity surface area in 2050s. Additional File 2: Supplement Figure S5. Multivariate environmental similarity surface and most dissimilar variable analysis under different combinations of climate change scenarios in 2070s. a Multivariate environmental similarity surface and most dissimilar variable in 2070s. b Heat map of multivariate environmental similarity surface area in 2070s. Additional File 2: Supplement Figure [file 43897_2024_136_MOESM2_ESM.zip › Supplementary Figures/Fig.S8.tif]

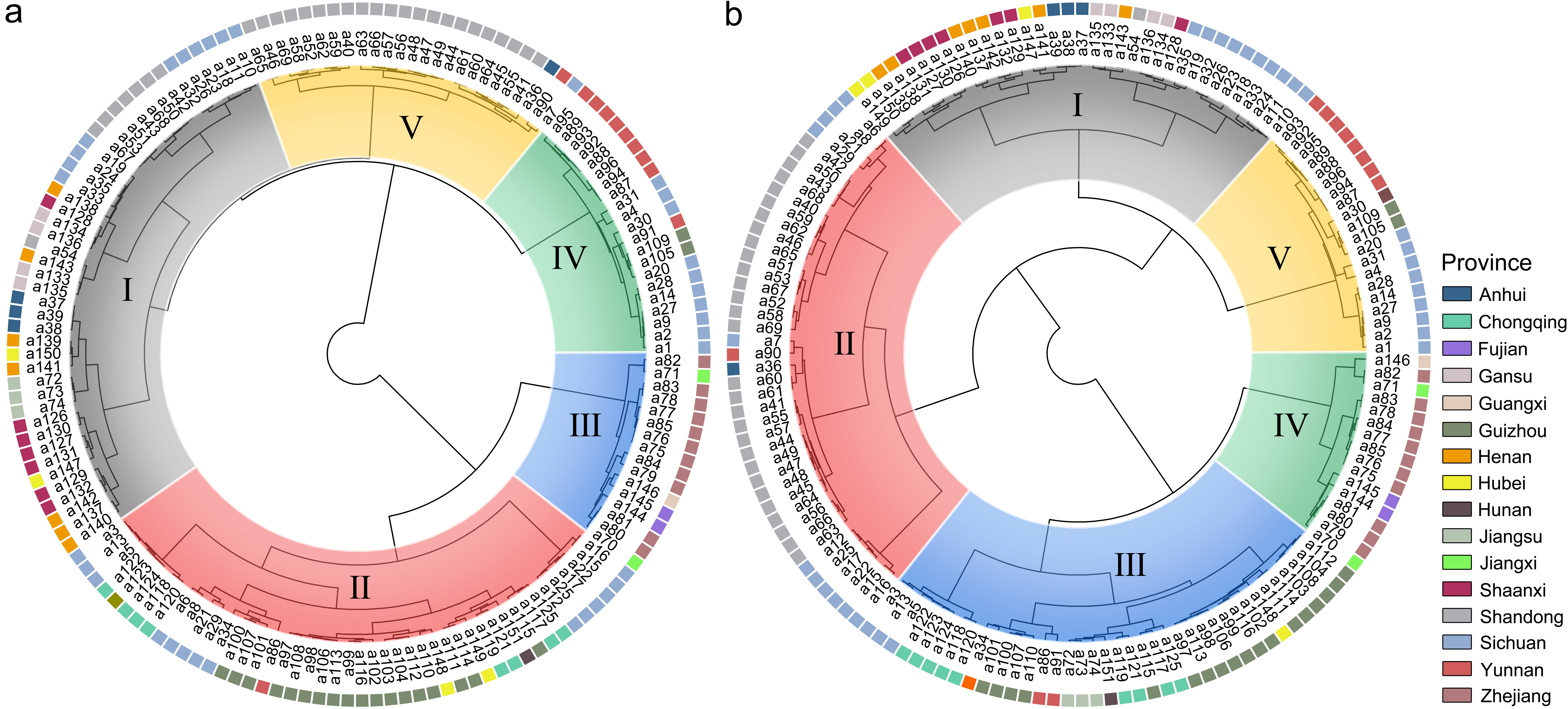

Supplement: Supplementary file 2 — Additional File 2: Supplement Figure S1. The landform with 151 distribution records of P. pseudocerasus. a Elevation. b Slope. c Aspect. Additional File 2: Supplement Figure S2. Ecological niche comparisons of P. pseudocerasus under different pathways in 2050s. a Ecological niches of P. pseudocerasus with SSP126-2050s–SSP585-2050s. Red arrows indicate Schoener's D. Blue indicates ecological niche overlap, green indicates unfilling, and red indicates expansion. b Alternative verification method of ecological niches with SSP126-2050s–SSP585-2050s. c Ecological niche similarity of different pathways in 2050s. The red arrow indicates the centroids of each species' realized ecological niche. Additional File 2: Supplement Figure S3. Ecological niche comparisons of P. pseudocerasus under different pathways in 2070s. a Ecological niches of P. pseudocerasus with SSP126-2070s–SSP585-2070s. Red arrows indicate Schoener's D. Blue indicates ecological niche overlap, green indicates unfilling, and red indicates expansion. b Alternative verification method of ecological niches with SSP126-2070s–SSP585-2070s. c Ecological niche similarity of different pathways in 2070s. The red arrow indicates the centroids of each species' realized ecological niche. Additional File 2: Supplement Figure S4. Multivariate environmental similarity surface and most dissimilar variable analysis under different combinations of climate change scenarios in 2050s. a Multivariate environmental similarity surface and most dissimilar variable in 2050s. b Heat map of multivariate environmental similarity surface area in 2050s. Additional File 2: Supplement Figure S5. Multivariate environmental similarity surface and most dissimilar variable analysis under different combinations of climate change scenarios in 2070s. a Multivariate environmental similarity surface and most dissimilar variable in 2070s. b Heat map of multivariate environmental similarity surface area in 2070s. Additional File 2: Supplement Figure [file 43897_2024_136_MOESM2_ESM.zip › Supplementary Figures/Fig.S9.tif]
